# Evidence of Population Fragmentation of the Western European Hedgehog *Erinaceus europaeus* in Urban Landscapes

**DOI:** 10.1002/ece3.73489

**Published:** 2026-04-22

**Authors:** Jessica Turner, Chris Carbone, Becki Lawson, Katharina Seilern‐Macpherson, Chris G. Faulkes

**Affiliations:** ^1^ Institute of Zoology, Zoological Society of London London UK; ^2^ School of Biological and Behavioural Sciences Queen Mary University of London London UK

**Keywords:** connectivity, fragmentation, hedgehogs, landscape genetics, population genetics, urban

## Abstract

Genetic diversity and connectivity are critical for the long‐term viability of wildlife populations but may be compromised in severely fragmented urban environments. Western European hedgehogs ( *Erinaceus europaeus* ) have undergone severe population declines but appear to thrive in urban environments. However, the species' vulnerability to fragmentation within these landscapes is not well understood. Here, we investigate the genetic structure and connectivity of Western European hedgehogs in Greater London, UK, and surrounding areas using 1413 single nucleotide polymorphisms from targeted sequencing of ultraconserved elements. Across the study region, we found limited genetic structuring except for within Greater London, where one highly urbanised site was clearly differentiated. Individual genetic diversity showed only some evidence of a negative relationship with the proportion of surrounding urban land cover. However, populations within Greater London were found to exhibit lower genetic diversity and higher differentiation in three of four populations investigated compared to four populations from outside the city, consistent with the assumption of greater fragmentation and elevated drift. For hedgehogs within Greater London, landscape connectivity modelling identified that habitat resistance better explained observed genetic differentiation than distance, but this was not seen in suburban hedgehogs separated by agricultural landscapes outside the city. These results present important evidence that hedgehog populations occupying highly urban environments within cities are vulnerable to genetic isolation due to habitat fragmentation, with implications for our understanding of the role of urban environments in supporting hedgehog populations. Moreover, these findings contribute to the broader understanding of the long‐term persistence of native wildlife in these landscapes.

## Graphical abstract

The Western European hedgehog ( *Erinaceus europaeus* ) is a declining mammal increasingly associated with urban landscapes. This study uses 1413 SNP markers to investigate the species' genetic structure and connectivity in Greater London, UK, and surrounding areas. We identify evidence of genetic isolation in highly urban populations, despite little genetic structure across the wider region. This work contributes to knowledge of the threats facing urban hedgehogs, and to broader understanding of the long‐term persistence of native wildlife in urban areas.

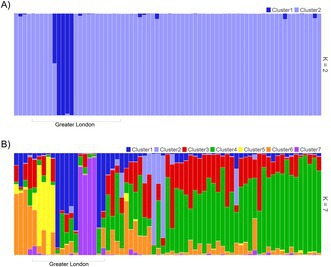

## 1 Introduction

The rapid growth of urbanisation is considered a significant threat to biodiversity globally (Piano et al. 2020; Simkin et al. 2022), as natural habitat is converted to human settlements dominated by dense built infrastructure (Johnson and Munshi‐South 2017). In severely fragmented urban landscapes, wildlife restricted to diminished greenspaces habitats may have smaller, more isolated populations that are more susceptible to decline. In particular, small populations are at risk of elevated genetic drift, leading to the loss of genetic diversity over time and reduced capacity to adapt to environmental change (Freeland et al. 2011), as well as inbreeding depression and lowered population fitness due to limited availability of unrelated mates (Kardos et al. 2016).

There is widespread evidence of urbanisation impacts on drift and gene flow in wildlife populations (Miles et al. 2019). Mammals across North America, for example, have been found to have reduced population size and genetic diversity, and higher differentiation in populations more heavily impacted by urbanisation, which may exacerbate the pressures they face in these environments (Schmidt et al. 2020).

Gene flow, in which individuals move between populations to breed, can alleviate the effect of small population size (Frankham 2015). However, species movement can be strongly impacted in complex urban landscapes (LaPoint et al. 2015) by abrupt landcover changes at small spatial scales, combined with larger scale gradients from increasing densification of built infrastructure and disturbance with proximity to the urban core (McKinney 2008). Urban landscapes can pose both physical (Beninde et al. 2016) and behavioural barriers (Kimmig et al. 2020) for wildlife movement, which can be location and species‐specific. In brown rats (*Rattus norvegicus)*, genetic discontinuities within urban areas were driven by low resource availability in a central commercial district in New York, by major waterways in New Orleans, and by roads in Vancouver and Salvador (Combs, Byers, et al. 2018; Combs, Puckett, et al. 2018). In contrast, tree canopy cover was found to facilitate gene flow in white‐footed mice (*Peromyscus leucopus)* in New York City parklands (Munshi‐South 2012).

As habitats within urban landscapes can support diverse communities of native wildlife (Parsons et al. 2018), including species of conservation concern (Boakes et al. 2024; Soanes and Lentini 2019), conserving urban biodiversity is a growing priority (Dearborn and Kark 2010). Understanding the potential role of genetic processes in impacting the long‐term persistence of urban wildlife populations, therefore, will be valuable to help support biodiverse cities in the future.

One species of conservation concern which is increasingly associated with urban landscapes is the Western European hedgehog ( *Erinaceus europaeus* ; hereafter hedgehog), where the species can reach high population densities (Hubert et al. 2011; Schaus et al. 2020). Though found across much of Europe and once considered common, the species has undergone declines across its range and was recently moved to Near Threatened in the IUCN Red List (Gazzard and Rasmussen 2024). In Britain, decline has been particularly severe in rural populations, whereas hedgehogs in urban areas have experienced a milder decline and show signs of recovery (Wembridge et al. 2022). Potential factors contributing to the species' relative success within urban environments include the availability of natural prey and supplemental feeding by householders (Gimmel et al. 2021; Hubert et al. 2011), sheltered environmental conditions (Pettett et al. 2017), as well as lower predation pressure (Van De Poel et al. 2015).

Habitat loss and fragmentation are considered important threats for hedgehogs, with agricultural intensification seen as a major contributor to their decline in rural areas (Williams et al. 2018). The impact of habitat fragmentation in urban landscapes, however, is not well understood. As a terrestrial mammal with small urban home ranges (Rasmussen et al. 2019; Reeve 1982) and reliance on greenspace habitats (Hof and Bright 2009), hedgehogs may be at risk of increasing urban densification (Taucher et al. 2020) and barriers such as high fences (Hof and Bright 2009), roads (Rondinini and Doncaster 2002; Wright et al. 2020), or busy transport networks and rivers (Braaker et al. 2014) prevalent in urban landscapes. On the other hand, as a behaviourally flexible ‘synanthropic’ species, able to use anthropogenic habitats such as domestic gardens and parks, hedgehogs may be able to maintain connectivity in fragmented urban landscapes. In Berlin, hedgehogs were found to cope with permanent habitat fragmentation at the local scale by increasing their home range size (Berger et al. 2020).

Previous studies have found evidence of local genetic fragmentation among hedgehog populations in Oxfordshire, UK (Becher and Griffiths 1998), and at larger scales in Denmark (Rasmussen et al. 2020) and in the Iberian Peninsula (Araguas et al. 2022). Within cities, findings have been mixed, with high genetic diversity and gene flow found in Berlin (Barthel et al. 2020) whilst genetic clustering was identified in Zurich (Braaker et al. 2017). However, these studies did not include comparison to the surrounding landscape context. Low diversity and a lack of geographic structure was found in hedgehogs in Helsinki and surrounding regions, though possibly due to recent founder events (Osaka et al. 2022), whilst an investigation across South Wales that included suburban and urban areas found no relationship between hedgehog genetic structure and potential barriers, landscape resistance or distance (Shove et al. 2025). In contrast, hedgehogs in a zoo in Barcelona were found to be differentiated from surrounding areas, suggesting genetic isolation in a highly urban population (Araguas et al. 2022).

Eastern England contains some of the highest relative hedgehog abundance in Britain (Wembridge et al. 2022), making it an important region in which to investigate hedgehog conservation status. Greater London, in southern England, is the largest urban landscape in the UK covering 1572 km^2^ (Office for National Statistics, ONS, 2020), bisected by the river Thames. Although hedgehogs were historically found across the city (Morris 1966), they have declined in the inner suburbs and been largely lost from the city's core, except for Regent's Park, which is now considered to be the most central population in the city (Gurnell et al. 2024; Morris 2018).

Previous habitat suitability modelling has suggested that increasing urban pressure and loss of vital greenspace habitats such as parks, gardens and allotments with proximity to the city's core has contributed to the decline of hedgehogs in Greater London (Fung et al. 2024; Turner et al. 2022). Furthermore, using mitochondrial DNA to investigate phylogeographic diversity in hedgehogs across Greater London, east and south‐east England identified some evidence of lower local haplotype diversity within the city despite widespread distribution of haplotypes across the study area (Turner et al. 2025). However, the capacity of these mitochondrial markers to elucidate fine‐scale contemporary genetic structure is limited, and there is a need to more clearly understand whether hedgehogs that persist within the city are experiencing genetic fragmentation.

The present study uses a higher resolution genomic dataset of 1413 single nucleotide polymorphisms identified through targeted sequencing of ultraconserved elements (UCEs) to investigate fine‐scale genetic structure of hedgehogs in Greater London and surrounding regions. UCEs are highly conserved sequences found throughout the genomes of many taxa and are widely used to address phylogenetic questions involving deep‐time divergences (Faircloth et al. 2012). Whilst the core UCE sequence is highly conserved, substitutions can accumulate in flanking regions which are suitable for investigating divergences at more recent timescales. The utility of UCEs to investigate population structure and gene flow at the inter‐species (Winker et al. 2018) and intra‐species (Mapel et al. 2021; Nikolakis et al. 2022; Parker et al. 2020; Stiller et al. 2021) levels is being increasingly explored.

Across the study area, we investigate (i) if hedgehogs exhibit genetic structuring, predicting that if hedgehogs are poor dispersers, there will be clear geographic structuring across the region and (ii) if the genetic diversity of individuals is related to the surrounding proportion of urban landcover, with the expectation that individuals in highly urban locations will have lower genetic diversity. Using a subset of data from eight populations in two contrasting areas, from four sites within Greater London and four locations outside the city, we compare genetic diversity and differentiation to (iii) identify whether populations within the city show lower genetic diversity and higher differentiation consistent with urban fragmentation. Within the same two areas, we explore whether (iv) isolation by distance or the landscape resistance better explains observed genetic differentiation among hedgehogs.

## 2 Methods

### 2.1 Sample Collection

Tissue samples from deceased hedgehogs were collected from 2012–2022 from Greater London (65), and surrounding counties of Hertfordshire (40), Cambridgeshire (6), Surrey (3), Kent (2), Essex (2), Bedfordshire (1), Suffolk (1), Berkshire (1), and four samples of unknown origin. Samples were provided by the Garden Wildlife Health project (56), a national wildlife disease surveillance scheme, from the −20°C tissue archive housed at ZSL (2012–2020), as well as from rescue centres (38), and were opportunistically collected by volunteers (e.g., from parks, roadkill) (31). Date of collection, sex (if known), and location coordinates were recorded. For samples where only the 1 km Ordnance Survey (OS) grid reference was available (26), coordinates at the grid cell centre were used. Individuals were grouped by location if they were found within approximately 1 km of each other, therefore within the distance that hedgehogs are known to travel per night (Morris 1988) and potentially from the same population and, for samples outside of Greater London, were within the same town or village. Assignment to these broader localities enabled recognition of clustering in the distribution of samples in the dataset.

### 2.2 DNA Library Preparation

Sample storage and DNA extraction were conducted as described in Turner et al. (2025). Dual‐index libraries were prepared using the NEBNext Ultra II FS DNA Library Prep Kit for Illumina (New England BioLabs Inc., Ipswich, MA, USA) following the manufacturer's recommended protocol for DNA inputs ≥ 100 ng. The size selection protocol was carried out using AMPure XP Beads with conditions to retain fragment insert sizes of 375–475 bp, which is suitable for use with UCE libraries (*pers. comm* Daicel Arbor Biosciences, Ann Arbor, MI, USA). Half volumes of reagents were used throughout. Samples with high DNA concentration were standardised to 500 ng of input DNA. For low DNA concentration samples, all available DNA was used and concentrated through dehydration to 13 ul before library preparation. Final undiluted libraries were assessed using a D1000 Agilent Tapestation (Agilent Technologies) to check library concentration and size distribution.

Libraries were successfully prepared for 109 samples, which were pooled in equimolar proportions into 13 pools and dehydrated before shipping to Daicel Arbor Biosciences, Ann Arbor, MI, USA for target enrichment using the mybaits UCE Tetrapods 5Kv1 baits kit following the standard mybaits v5.02 Protocol, with overnight washes and washes at 65°C. Captures were pooled in approximately equimolar ratios and sequenced on an Illumina NovaSeq 6000 platform on a partial S4 PE150 lane to approximately 1 Gpb per library.

### 2.3 Bioinformatic Processing

The raw demultiplexed reads were quality checked using FastQC (Andrews 2010) and cleaned via the illumiprocessor function in Phyluce 1.7.1 (Faircloth 2016). Cleaned reads were mapped to the reference EriEur 2.0 genome (GCA_000296755.1) using Burrows Wheeler Alignment in bwa V 0.7.17 (Li and Durbin 2009). Output files were sorted by name in Samtools V 1.1 (Danecek et al. 2021), read pairing information cleaned, and duplicates removed. Qualimap V.2.2.1 (Okonechnikov et al. 2016) was used to analyse the reads mapped with a quality score of 20 or higher.

Selected files were viewed in IGViewer (Robinson et al. 2011), identifying that some reads mapped away from the target UCE loci. To exclude these, the UCE probe set was downloaded from the Faircloth lab GitHub (https://raw.githubusercontent.com/faircloth‐lab/uce‐probe‐sets/master/uce‐5k‐probe‐set/uce‐5k‐probes.fasta) and mapped to the reference hedgehog genome. The output file was sorted in Samtools, compressed, and converted to bed format using the ‘bam2bed’ function in bedtools V.2.28 (Quinlan and Hall 2010). The Bedtools ‘slop’ function was used to extend the region around each mapped UCE probe by 1000 bp in each direction, which was deemed sufficient to include all reads mapping around the UCE probe through visual inspection in IGViewer. Samtools ‘view’ function was used to retain reads within these UCE regions only, which were then sorted, indexed, and UCE‐specific depth information extracted using the Samtools ‘depth’ function.

Variant calling was conducted using bcftools ‘mpileup’ (Danecek et al. 2021) set to include only SNPs. Information tags were filled with bcftools ‘filltags’ function and files converted to VCF format for further filtering in VCFtools (Danecek et al. 2011). It has been recommended that individuals with high levels of missing data be removed before further filtering (Cerca et al. 2021), missing data statistics were calculated for individuals and loci. Seven individuals with more than 20% missing data, and one with unusually high heterozygosity, were removed. Further filtering was carried out in BCFtools, guided by recommendations of O'Leary et al. (2018) with some adjustments. Loci with SNP calls supported by genotype read depths less than 5, quality scores less than 20, mean read depth per locus across individuals less than 10, and mapping quality less than 30 were removed (filtering results are shown in Table S1). Sites with a minor allele count (MAC) below three were also removed.

Sites genotyped for fewer than 95% of individuals were removed, and remaining sites restricted to include only biallelic SNPs. These were further thinned to select only one SNP per UCE locus to prevent inclusion of closely linked SNPs in the dataset. Final statistics for the filtered dataset were obtained using VCFtools and visualised by density plots and histograms plotted in R version 4.3.0 (R Core Team 2023) (Figure S1). One sample had high missing data following filtering and was therefore also removed prior to downstream analysis to leave 100 samples.

### 2.4 Environmental Data

Environmental landcover data was primarily sourced from Ordnance Survey vector landcover maps, and landcover classes of interest extracted in QGIS V 3.16 (Hannover, QGIS Development Team 2020). Open Street Map (OSM) queries were also used to identify farmland, wetland, and car‐parking landcovers using the QuickOSM plugin v 2.1.1 (Trimaille 2023) in QGIS (full landcover details and sources are given in Table S2). Linear features (roads, railways, and linear waterways) were buffered with minimum 5 m buffers to ensure these features were not lost when converted to grid format, before all landcover layers were converted to 5 m raster format in QGIS. These were aligned and combined into a single layer using the SAGA ‘Mosaicking’ tool. As landcovers could overlap, for example, a pond within a park, an order of priority (Table 1) was assigned so that the value from the first landcover encountered was used when constructing the mosaic layer. Buildings were set to highest priority whereas landcovers such as parks and gardens were set to lower priority, as they often contain other features such as waterways, paths, and lawns. Due to computational limitations, landcovers were resampled to 100 m resolution and only mosaiced within 3 km buffers of each sample at the regional scale with a 10 m interpolation distance for use in individual genetic diversity analysis. For generating resistance layer maps for local scale connectivity analysis in two contrasting areas, a 5 m resolution and 1 m interpolation distance was used, with the extent of the areas set to a minimum 3 km distance from the samples to reduce the impact of artificial map boundaries (Braaker et al. 2014).

**TABLE 1:** Table showing the landcover variables used in resistance modelling of Western European hedgehog *Erinaceus europaeus* .

Habitat variable	EMP resistance layer
Buildings	ABS
Path	14
Waterbodies	ABS
Railways	100
Small street	8
Highways	100
Large street	100
Linear Water	100
Wetland	100
Impervious	13
Woodland	100
Lawn	5
Amenity	6
Parks and Playspace	1
Cemeteries	1
Allotments	1
Farmland	100
Private Garden	1
Unclassified	15

*Note:* Landcover types are shown in descending order of precedence such that when landcover types overlap (e.g., a path within a park) the value of the landcover that is higher in the table is retained. A value of 1 indicates low, and 100 high, resistance to movement, and ‘ABS’ stands for an absolute barrier to hedgehog movement. In the isolation by distance model (NULL), all landcovers are set to a resistance of 1, in which genetic distance increases with linear distance. EMP indicates the empirical model of isolation by resistance, in which landcovers are assigned resistance values to movement for urban hedgehogs based on literature (Braaker et al. 2014; App et al. 2022).

### 2.5 Identification and Removal of Relatives

The presence of highly related individuals can bias population genetic inferences (O'Connell et al. 2019; Wang 2018). Pairwise relatedness among samples was calculated using the Queller and Goodnight (QG) estimator in Coancestry software (Wang 2011) and assessed using the R package ‘related’ (Pew et al. 2015), which was identified as a suitable estimator for the data through simulations (see Methods S1). Locus error rates were calculated in the R package poppr (Kamvar et al. 2014) whilst genotyping error rate and allelic drop‐out were unknown and set to zero throughout.

The Regent's Park was identified as being over‐represented in the dataset with 33 samples spanning 2013–2021. This site has been the subject of long‐term monitoring and hedgehog post‐mortem examinations with sample collection regularly conducted by the Garden Wildlife Health project. These samples also showed high relatedness, with 13 first order relative pairs, and likely comprised several generations of family members (see Figure S2 for pairwise relatedness plots). To reduce the effect of the oversampling and high relatedness identified at this site, samples from this location were subset to include only the five most recent samples (2019–2021), excluding pairs of first‐degree relatives. This resulted in a final dataset of 72 samples for analysis in this study.

## 3 Analysis

### 3.1 Spatial Genetic Structure

Spatial genetic structure across the study area (*n* = 72) was assessed using the Bayesian Markov Chain Monte Carlo (MCMC) algorithm implemented in STRUCTURE version 2.3.4 (Pritchard et al. 2000), to assign individuals in a dataset to a predefined number of discrete populations (*K*) (Porras‐Hurtado et al. 2013). Separate population priors were used to account for uneven sampling between locations (Wang 2017) with 10,000 burn‐in and 10,000 MCMC repetitions, for values of *K* ranging from 1–10 and five repetitions of each. The optimal *K* value was estimated using the Evanno method (Figure S3) (Evanno et al. 2005) and proportion of individual assignment to population clusters visualised using the R package ‘pophelper’ (Francis 2017). An initial *K* = 2 was identified, with one location (*n* = 5) forming a separate cluster from the rest of the dataset.

The presence of further hierarchical population structure was assessed by removing the location that formed a separate cluster and rerunning STRUCTURE on the remaining 67 samples, as it has been suggested that the software can be biased towards *K* = 2 (Janes et al. 2017). For comparison, a Principal Components Analysis (PCA), which has fewer assumptions, was also conducted in R using adegenet (Jombart 2008) to visualise genetic variation in the study area.

### 3.2 Individual Genetic Diversity Along the Urban Gradient

To relate hedgehog genetic status to urbanisation, individual genetic diversity measures were calculated for all samples (*n* = 72) and correlated with the proportion of urban landcover in surrounding 1 km and 3 km buffers. The number of heterozygous sites was calculated by subtracting the number of homozygous sites from the total number of loci sequenced for each individual, obtained via the VCFtools ‘het’ function. This was used to identify the proportion of heterozygous sites for each individual and divided by the mean heterozygosity across all individuals to give individual standardised heterozygosity (Hs) (Coltman et al. 1999).

To calculate the proportion of urban landcover around each individual, landcovers were first reclassified so that urban features (buildings, road types, railways, and impervious cover) had a value of 1, and other landcovers (greenspace types, water, and farmland) had a value of 0. The proportion of urban landcover in 1 km and 3 km buffers around sample locations was then calculated using the ‘zonal statistics’ tool in QGIS. Linear models in R were constructed with Hs as the response and proportion urban landcover in each buffer class as the predictor variable. Spatial autocorrelation in the model residuals was assessed by calculating overall and local Morans I in the sdpep package in R (Pebesma and Bivand 2023). Visualisation of spatial patterns of autocorrelation using ggplot2 (Wickham 2016) identified strong local spatial autocorrelation in individuals at the Regent's Park site. As for the clustering analyses, models were rerun with this site excluded (*n* = 67) to investigate this location's impact on the results, and spatial autocorrelation re‐assessed.

### 3.3 Population Genetic Diversity and Differentiation

**FIGURE 1:**
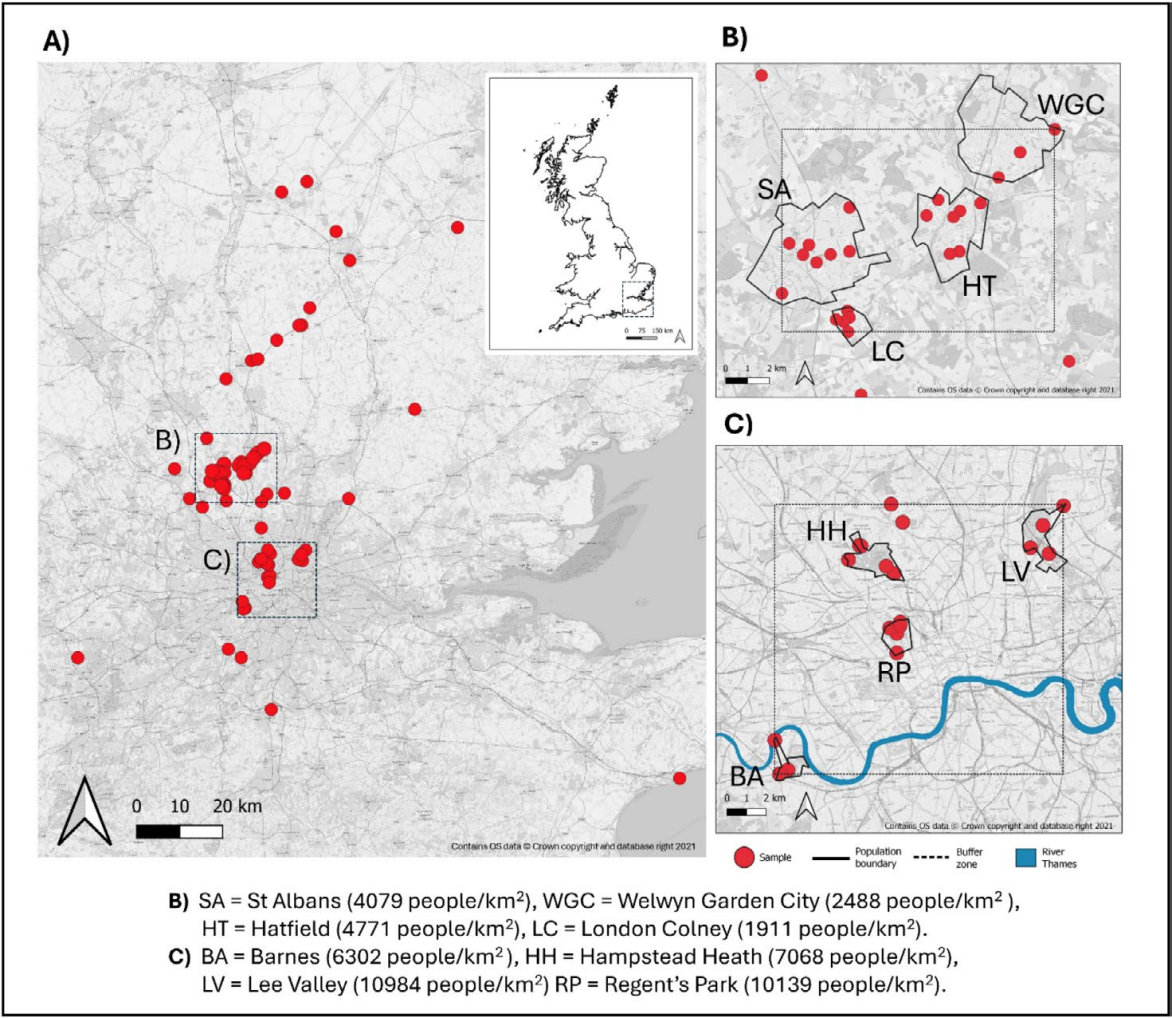
(A) Map showing sample locations (*n* = 72) of Western European hedgehog *Erinaceus europaeus* from east and south‐east England. The wider location of the study region is shown in the inset map, and data subsets used in population genetic and landscape connectivity modelling outlined in boxes (B) and (C) for ‘Outside London’ (in Hertfordshire) and ‘Within Greater London’, respectively. Population boundaries are outlined with solid black lines. Populations in box (B) are St Albans (SA), Welwyn Garden City (WGC), Hatfield (HT), and London Colney (LC). Populations in Box (C) are Hampstead Heath (HH), The Regent's Park (RP), Barnes (BA) and Lee Valley (LV). Two additional samples in Box (C), not assigned to populations, were also included in the landscape connectivity analyses only. The River Thames is shown in blue in box (C). Population codes are displayed below the map, alongside mean human population density values calculated from Middle Super Output Areas (ONS, 2021) as an indication of relative urbanisation levels. Details on mean human population density calculation are provided in Table S3.

Population genetic parameters were calculated for eight localities from which multiple samples (*n* ≥ 3) were sourced. These included four locations within Greater London and four locations in Hertfordshire, outside of the city (Figure 1), representing a range of relative urbanisation levels (Table S3). Due to bias in the source of individuals towards urban areas, all populations outside of Greater London in Hertfordshire were in smaller towns and one city, separated by agricultural landscape. The number of private alleles in each population was calculated in poppr (Kamvar et al. 2014), and allelic richness rarefied to the smallest sample size (*n* = 3) calculated in hierfstat (Goudet and Jombart 2022). Observed heterozygosity (Ho), expected heterozygosity (He), and inbreeding (Fis) were calculated in adegenet (Jombart 2008). Pairwise Wilcoxon rank sum tests with Benjamini‐Hochberg correction for multiple comparisons (Benjamini and Hochberg 1995) were used to identify significant differences in allelic richness, Ho and He among populations. Population differentiation was examined by calculating the overall and pairwise Weir and Cockerhams population Fst in hierfstat (Goudet and Jombart 2022), and the pairwise Fst results plotted using pheatmap (Kolde 2025).

### 3.4 Landscape Genetics

Connectivity was assessed for two contrasting areas in the study region, to compare gene flow patterns within and outside of Greater London (Figure 1b,c). These two areas, which contained the populations used in the population level analysis, were selected as they contained the greatest density of samples. Two additional samples within the area examined in Greater London but not part of a population were also included, to maximise the sample size for pairwise connectivity calculations. The two areas were therefore designated as ‘Greater London’, containing 20 individuals over 6 locations, and ‘Outside Greater London’, containing 24 individuals in 4 locations. Due to the highly heterogenous nature of urban landscapes, high resolution landcover maps were used (5 × 5 m), which prevented the investigation of gene flow over larger areas due to computational demands.

The landcover map for each area was converted into a resistance surface, which represents the landscape as a grid where each cell is assigned a cost value based on its environmental characteristics that represents the ease with which organisms are expected to traverse the cell. Assigning resistance values can be challenging (Peterman and Pope 2021) and here, we use resistance values previously parameterised for urban hedgehogs via radiotracking of hedgehogs in Zurich (Braaker et al. 2014) (Table 1). Railways and farmland were not present in Braaker et al. (2014), and were set to the same high resistances used in App et al. (2022). Unclassified landcovers in the study area consisted of a range of land uses and were set to be slightly more resistant than impervious surfaces for hedgehogs, to avoid being artificially viewed as barriers (by setting to maximum resistance) or corridors (by setting to minimum resistance). Buildings and large waterbodies were set to be impassable. This resistance layer was designated the empirical model of isolation by resistance (EMP). An undifferentiated resistance layer where all cells had uniform resistance to movement was also created as a model of isolation by distance (NULL), where movement cost increases linearly with distance (Row et al. 2017).

Single variable resistance surfaces were also explored for selected landcovers hypothesised to potentially impact hedgehog movement. Roads and water were tested as barriers, greenspace as corridors, and farmland and railways as either barriers or corridors for hedgehogs. This was done by setting the landcover of interest to either 1 (low resistance to movement) or 100 (high resistance to movement), and other landcovers set to either 1 or 100 in the opposite direction (Table 2).

**TABLE 2:** Table showing the values for single landcover surfaces to explore the impact of specific landcover features on Western European hedgehog *Erinaceus europaeus* connectivity.

Single landcover layer	Landcover resistance	All other landcovers**
RAILBAR	100	1
RAILCOR	1	100
ROADBAR	100	1
GREENCOR	1	100
FARMCOR*	1	100
FARMBAR*	100	1
WATERBAR	100	1

*Note:* A value of 1 indicates low, and 100 high resistance to movement. BAR and COR refer to the landcover as a barrier and corridor respectively; RAILBAR for example indicates railways as high resistance barriers whereas RAILCOR indicates railways as low resistance corridors for movement and all other habitats as barriers. * Indicates hypotheses that were only tested outside of Greater London due to lack of landcover type within the city. ** Buildings and waterbodies were always parameterised as absolute resistance to hedgehog movement.

Two algorithms implemented in the ‘gdistance’ package in R (Van Etten 2017) were used to model gene flow. These were least cost paths, using the ‘costDistance’ function, and random walk commute distance, using the ‘commuteDistance’ function. Least cost path analysis identifies the shortest route of lowest cumulative resistance to movement between two locations (Balbi et al. 2019) and can be used to identify critical movement corridors in the landscape. In contrast, the random walk approach to connectivity modelling considers all possible routes between locations, giving insight into areas of high movement probability (Dickson et al. 2019). Before running the algorithms, resistance surfaces were converted to the required input format, a graph formatted ‘transition layer’ that represents the likelihood of movement between neighbouring cells. In addition, a small value of 0.0001 was added to adjacent cells before calculation of commute distances, as the function requires that there are no clusters of isolated cells.

Euclidean genetic distance was calculated between all sample pairs in each area (20 samples in Greater London, 24 samples outside London) using the ‘dist’ function in adegenet (Jombart 2008). Individual‐based Maximum Likelihood Population Effects (MLPE) models were used to relate genetic and resistance distances using the corMLPE package (Clarke et al. 2002; N. Pope 2024), which accounts for the non‐independent error structure in pairwise datasets (Row et al. 2017). Genetic distance was fitted as the response variable and the log‐transformed resistance distance, either from the least cost path or commute distance algorithms, was fitted as the predictor variable, with individuals as the correlation structure. Each model was also run with a term for Euclidean distance, calculated using the undifferentiated landscape, to account for the effects of distance and reduce false significant associations of genetic distance with landscape variables (Row et al. 2017).

Models were compared using Akaike Information Criterion (AICc corrected for small sample size) and Bayesian Information Criterion (BIC), as recommended by Row et al. (2017), selecting the model with the lowest AICc value as the best model. Model fit was inspected using plots of residuals.

Finally, the ’AtoB’ function in gdistance was used to map the least cost path routes between all pairs of points in each study area. The ’passage’ function was used to calculate the number of random‐walk passages between pairwise points, and averaged to generate maps allowing visual inspection of landscape connectivity patterns.

## 4 Results

### 4.1 Sample Collection

Overall, 125 hedgehog samples were collected (Table S4), of which DNA extraction and library preparation was successful for 109. Following UCE target‐capture, sequencing and bioinformatic processing, and visualisation of SNP quality, a dataset of 1413 SNPs was retained for 100 samples. To minimise the effects of biased sampling and related individuals in the dataset, an additional 28 samples were removed from one highly sampled location (The Regent's Park), to leave 72 samples for analysis. These were from within Greater London (22), Hertfordshire (37), Cambridgeshire (6), Surrey (2), Kent (1), Essex (2), Suffolk (1), Berkshire (1) (Figure 1a).

### 4.2 Spatial Genetic Structure

**FIGURE 2:**
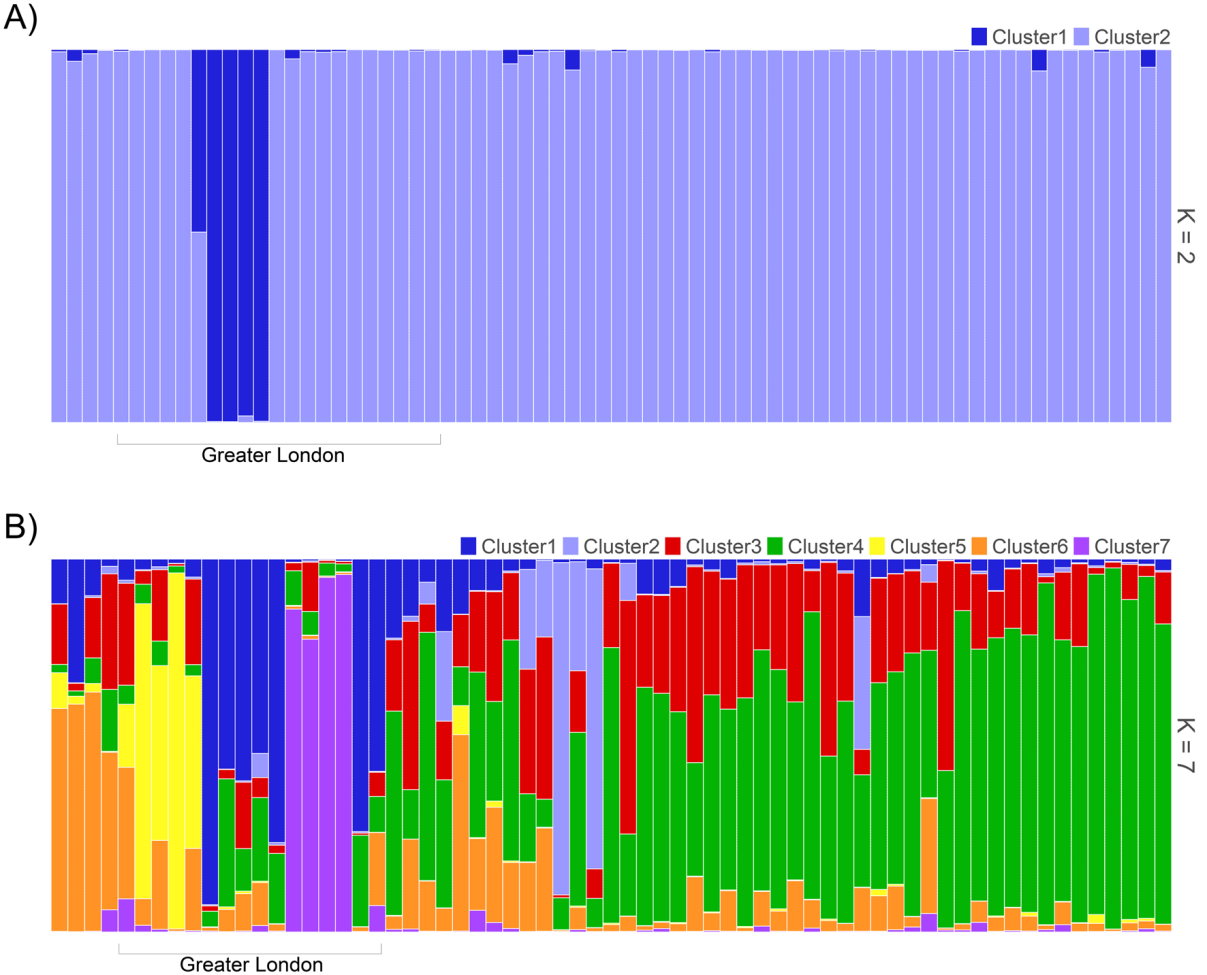
Population clustering of Western European hedgehog *Erinaceus europaeus* as assessed by STRUCTURE. (A) The full dataset (*n* = 72) was assigned *K* = 2. (B) Analysis of further substructure in Cluster 2 from initial analysis identified *K* = 7. Each vertical bar represents one individual, grouped by location and arranged by latitude so that south is on the left and north on the right. Clusters 1, 5 and 7 are found in Greater London, indicated in the Figure. Colours represent proportional assignment of individuals to populations.

Population clustering analysis using STRUCTURE identified *K* = 2 populations. One site, The Regent's Park, which is located close to the core of Greater London, formed a separate population despite being geographically central in the study region. After exclusion of this population to assess further substructure, *K* = 7 was identified, although the identified population structuring was weak. Population clustering broadly followed a latitudinal pattern, with one cluster prevalent in the north of the study area, and a second in the south, whilst individuals in the centre of the study region showed high levels of admixture among multiple populations (Figure 2). Notably, three clusters occurred predominantly within Greater London, where they formed geographically localised groups.

**FIGURE 3:**
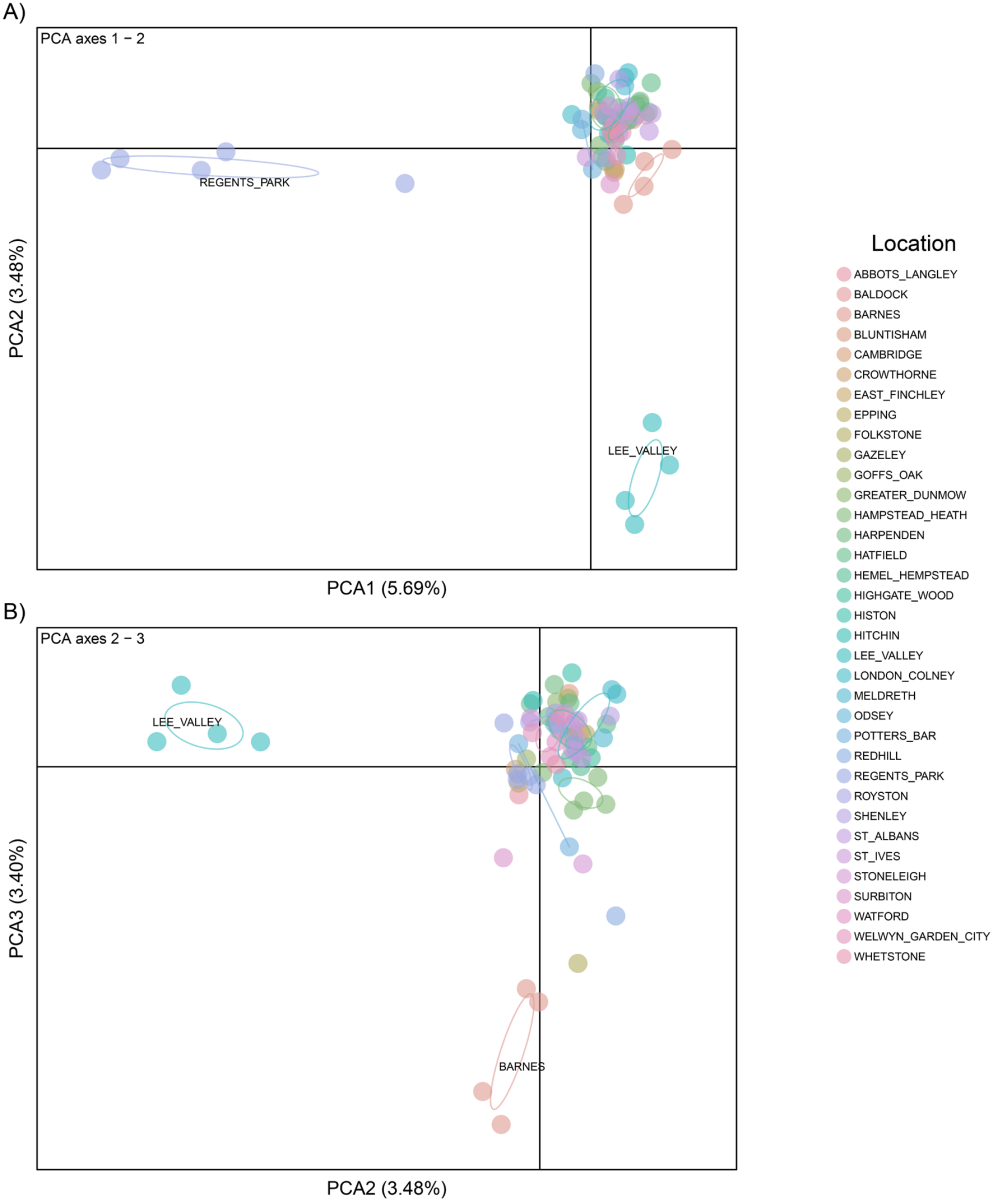
Principal Components Analysis of the Western European hedgehog *Erinaceus europaeus* dataset, (A) indicates the first and second axes, (B) indicates the second and third axes. Each point represents a single sample. Samples are coloured by general location with associated name.

PCA analysis supported the separation of The Regent's Park in the first axis of variation and detected separation of two further Greater London sites in the second axis of variation: Lee Valley and Barnes. However, most samples showed high overlap in their genetic variation, forming a single tight cluster (Figure 3).

### 4.3 Individual Genetic Diversity Along the Urban Gradient

Standardised heterozygosity ranged between 0.481–1.177. The proportion of urban landcover ranged from 0.051–0.571 (1 km) and 0.026–0.527 (3 km). When all samples were considered, a negative relationship between Hs and the proportion of urban landcover in the surrounding area was identified at both scales (1 km *p*‐value: 0.031, 3 km *p*‐value: 1.84e‐5). However, there was significant spatial autocorrelation in model residuals indicated by Morans I at the 1 km (*p*‐value: 0.0001692), though not at the 3 km scale (*p*‐value: 0.05218). Local Morans I indicated that spatial autocorrelation was mainly due to samples from The Regent's Park. When samples from this site were removed, this accounted for most spatial correlation in the dataset which became only marginally significant at the 1 km scale (1 km *p*‐value: 0.04847; 3 km *p*‐value: 0.0802). However, only a weakly significant negative relationship between Hs and proportion of urban landcover at the 3 km scale remained (*p* = 0.0213).

### 4.4 Population Genetic Diversity and Differentiation

Population summaries are shown in Table 3. Allelic richness is significantly lower in The Regent's Park (pairwise *p*‐values: < 2e‐16, all comparisons) and Lee Valley (pairwise *p*‐values: BA 0.0224, HT: 0.0209, SA: 0.0224, HH: 0.0036, LC: 0.0079), but significantly higher in Welwyn Garden City (pairwise *p*‐values:< 2e‐16, all comparisons), compared to the other populations. Significantly lower Ho was also identified in Barnes (pairwise *p*‐values: HT: 1.0e‐07, SA: 3.5e‐09, RP: 2.9e‐15, LV: 0.00011, HH: 0.01954, WGC: 6.3e‐05, LC: 0.00824), The Regent's Park (BA: 2.9e‐15, HT: < 2e‐16, SA: < 2e‐16, LV: 0.00178, HH: < 2e‐16, WGC: 3.9e‐16, LC: < 2e‐16), and Lee Valley (BA: 0.00011, HT: < 2e‐16, SA: < 2e‐16, RP: 0.00178, HH: 2.4e‐11, WGC: 1.5e‐12, LC: 2.7e‐12), and The Regent's Park also has lower He (pairwise *p*‐values: LV: 9.7e‐08, < 2e‐16 all other comparisons), compared to the other populations.

**TABLE 3:** Table showing population summaries for Western European hedgehog *Erinaceus europaeus,* for four populations within Greater London and four populations north of Greater London (Hertfordshire).

Dataset	Population	Sample size	Private alleles	Ar	Ho	He	Fis
Greater London	Barnes	4	40	1.532	0.230*	0.240	0.017
	Regent's park	5	63	1.391*	0.170*	0.180*	0.010
	Lee Valley	4	50	1.471*	0.200*	0.230	0.075
	Hampstead Heath	5	20	1.578	0.260	0.270	−0.010
Outside London	Hatfield	8	24	1.580	0.260	0.260	0.008
	St Albans	8	21	1.581	0.270	0.270	−0.012
	Welwyn Garden City	3	9	1.599*	0.270	0.270	−0.036
	London Colney	5	23	1.575	0.270	0.260	−0.039

*Note:* Sample size (*n*), number of private alleles, rarefied allelic richness (Ar), observed and expected heterozygosity (Ho and He), and inbreeding (Fis) are shown. * indicates Ar, Ho and He values which are significantly different to all other populations in pairwise comparisons (at the 5% level).

**FIGURE 4:**
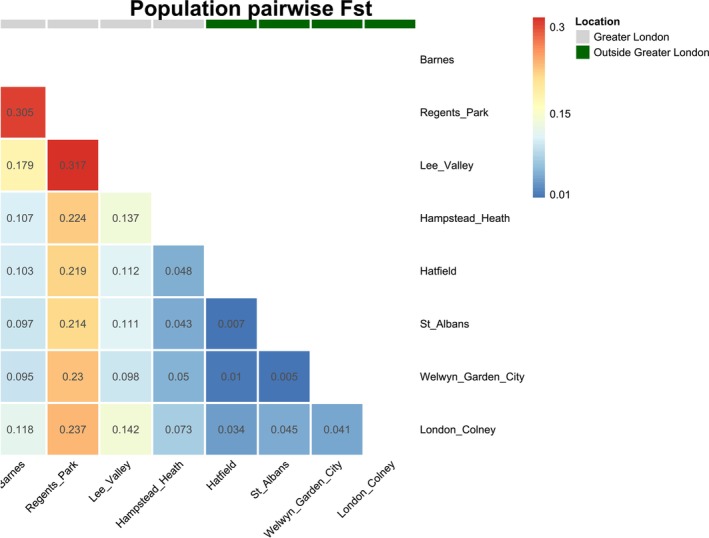
Heatmaps of pairwise Fst values between four populations of Western European hedgehog *Erinaceus europaeus* from Greater London and four populations from outside of Greater London (Hertfordshire). Blue indicates low Fst and red high Fst. Grey and green bars above the heatmap indicate the population as being from Greater London or outside of Greater London, respectively.

Overall, Fst among all populations was 0.1135 (0.1069–0.1206). Pairwise Fst (Figure 4) indicated high differentiation among three of the four Greater London populations (Barnes, The Regent's Park, and Lee Valley) and moderate differentiation of these from the populations outside of Greater London. In contrast, little differentiation was identified among the populations outside of Greater London. Hampstead Heath, although located within Greater London, also showed only low differentiation from populations outside of the city.

### 4.5 Landscape Connectivity

Within Greater London, the empirically parameterised resistance model outperformed isolation by distance using least cost path models, and in the commute distance model that included distance (Table 4). Interestingly, of the single landcover resistance models, models in which railways and greenspaces act as corridors to facilitate gene flow also outperformed isolation by distance.

**TABLE 4:** Table showing performance of least cost path (LCP) and random‐walk commute distance (COMM) models of landscape connectivity for Western European hedgehog *Erinaceus europaeus* in two contrasting landscapes: Greater London and Outside Greater London.

Area	Models	Hypothesis	AICc	*R*2	+DIST	AICc	*R*2
Greater London	LCP	NULL	993.782	0.459	NULL	993.782	0.459
		**EMP**	**981.382**	**0.514**	**EMP**	**980.427**	**0.513**
		RAILBAR	1002.581	0.455	RAILBAR	994.143	0.464
		RAILCOR	1039.718	0.326	RAILCOR	985.556	0.476
		ROADBAR	1004.998	0.462	ROADBAR	993.394	0.472
		GREENCOR	1009.571	0.435	GREENCOR	994.577	0.466
		WATERBAR	1006.677	0.439	WATERBAR	994.238	0.457
	COMM	**NULL**	1008.162	0.418	NULL	1008.162	0.418
		EMP	1036.746	0.426	**EMP**	**1002.985**	**0.458**
		RAILBAR	1031.427	0.415	RAILBAR	1008.494	0.429
		RAILCOR	1032.603	0.467	RAILCOR	1005.841	0.441
		ROADBAR	1042.624	0.369	ROADBAR	1008.567	0.430
		GREENCOR	1039.397	0.375	GREENCOR	1004.780	0.443
		WATERBAR	1043.652	0.368	WATERBAR	1009.329	0.417
Outside Greater London	LP	NULL	964.854	0.088	NULL	964.854	0.088
		EMP	977.568	0.073	EMP	966.423	0.093
		RAILBAR	966.261	0.104	RAILBAR	967.036	0.095
		RAILCOR	980.1167	0.051	RAILCOR	959.420	0.113
		ROADBAR	969.944	0.081	ROADBAR	967.199	0.088
		GREENCOR	980.491	0.057	GREENCOR	964.158	0.103
		FARMBAR	966.2771	0.111	FARMBAR	967.073	0.093
		**FARMCOR**	**650.753**	**0.144**	**FARMCOR**	**954.443**	**0.142**
		WATERBAR	964.735	0.097	WATERBAR	966.025	0.093
	COMM	**NULL**	**960.887**	**0.093**	NULL	960.889	0.093
		EMP	984.547	0.076	EMP	963.498	0.096
		RAILBAR	976.456	0.153	RAILBAR	962.058	0.110
		RAILCOR	967.515	0.242	RAILCOR	958.100	0.143
		ROADBAR	992.744	0.055	ROADBAR	963.634	0.098
		GREENCOR	978.726	0.134	GREENCOR	963.012	0.103
		FARMBAR	978.726	0.134	FARMBAR	963.246	0.093
		FARMCOR	967.584	0.340	**FARMCOR**	**955.047**	**0.184**
		WATERBAR	973.498	0.177	WATERBAR	961.320	0.118

*Note:* Akaike's Information Criterion adjusted for small sample size (AICc) and R‐squared (*R* ^2^) are shown for each. (+ DIST) indicates models including distance. The best performing model for each area and algorithm is highlighted in bold.

Outside of Greater London, the opposite pattern was observed, with isolation by distance outperforming landscape resistance. However, when accounting for geographic distance, the single landcover resistance models in which farmland and railways act as corridors did outperform the null model, suggesting a role for farmland and railways in facilitating hedgehog movement.

Visualisation of the least cost path routes of least resistance and cumulative random walk densities for the empirical model in Greater London highlighted that movement was concentrated in greenspace, such as gardens, whereas large roads and rivers appeared as barriers to movement. High overlap among the least cost paths between samples suggests limited availability of routes connecting hedgehogs in the study area; for example, only one bridge potentially connects hedgehogs in Barnes with populations north of the river Thames, which runs through the middle of the city.

## 5 Discussion

This study explores the impact of fragmented urban habitats on the Western European hedgehog, using 1413 SNP markers to investigate hedgehog population genetic structure and connectivity within Greater London and the surrounding region. We identified elevated genetic differentiation and reduced genetic diversity in three of four populations investigated within Greater London, consistent with fragmentation and genetic drift, compared to populations from suburban locations outside of the city. Landscape connectivity modelling revealed that resistance in the urban environment influences gene flow in hedgehogs within Greater London, but not in a contrasting landscape outside of the city. At the wider scale, we found some evidence of a trend towards lower genetic diversity in individuals with higher urban cover in the surrounding landscape, and only weak evidence of genetic structuring across the study region.

### 5.1 Spatial Genetic Structure

Spatial genetic structure was seen primarily within Greater London, with Bayesian clustering analysis initially identifying one site located centrally within Greater London, The Regent's Park, as a distinct cluster. Excluding this location, seven clusters were identified in the remaining dataset (*n* = 67). Although this structuring was generally slight, three of the seven further clusters identified were within Greater London, whilst samples from the wider study region showed varying levels of admixture and followed a generally latitudinal gradient in their population assignments. PCA analysis also distinguished individuals from sites within Greater London (particularly from Barnes, Lee Valley, and The Regent's Park), but found high genetic overlap among most remaining samples. These results suggest that hedgehogs maintain a connected population at the landscape scale, but that this may be disrupted within large urban centres.

An absence of strong landscape‐scale genetic structure has also been reported in hedgehogs from South Wales, where only weak genetic structuring and high levels of admixture were identified despite the presence of barriers such as roads and water (Shove et al. 2025). Previous findings from Zurich also identified population clusters within the city (Braaker et al. 2014), as seen in this study, whereas in Berlin hedgehogs were found to maintain gene flow, although with local clustering of related gamodemes (Barthel et al. 2020). Whilst hedgehogs in Helsinki were found to form a homogenous population across the city and surrounding area, it was uncertain whether this was due to a lack of landscape fragmentation or whether low genetic diversity remains from recent founder effects (Osaka et al. 2022). In contrast, Araguas et al. (2022) identified similar findings to our results, with a strongly differentiated highly urban zoo population, and some weaker evidence of differentiation between samples from Barcelona within Central Catalonia (Araguas et al. 2022).

### 5.2 Individual Heterozygosity and Urbanisation

Evaluation of the genetic diversity of individuals throughout the study area against the surrounding proportion of urban landcover found some evidence of lower Hs in locations with higher urban landcover, particularly at the 3 km scale, with a weakly significant negative relationship when samples from The Regent's Park site in central London were removed. These results suggest that for hedgehogs, genetic decline is associated with occupying a large urban landscape such as a city, where urban land covers may disrupt dispersal and gene flow that maintain genetic diversity. However, low Hs were also observed in individuals occupying landscapes with little urban landcover, suggesting that other factors may also influence individual genetic diversity.

Previous studies have shown little relationship between individual genetic diversity and urbanisation. (Richardson et al. 2021), for example, did not find a significant relationship between genetic diversity and urbanisation in native bats or mice in Providence, USA, despite recovering a positive relationship between inbreeding and urbanisation. In Denmark, hedgehog genetic diversity was found not to relate to farmland density, road density or human population density, except for a slightly significant negative association between inbreeding and farmland density (Rasmussen et al. 2020).

### 5.3 Population Genetics

Detailed comparison of populations within the highly urban landscape of Greater London and from smaller suburban locations north of the city enabled direct assessment of the impact of urban pressure on the genetic diversity and isolation of hedgehog populations. Ho among the eight populations examined ranged between 0.170–0.270. This is comparable to Danish hedgehogs where Ho between 0.124–0.293 was identified using a dataset of 2902 SNP markers (Rasmussen et al. 2020). The lowest Ho in this study was 0.170 in The Regent's Park in Greater London, whereas in Denmark the lowest Ho of 0.124 was observed in a small, isolated island population (Rasmussen et al. 2020).

Notably, three of the four populations within Greater London had significantly lower Ho (Barnes, Lee Valley, The Regent's Park) compared to the other populations examined, combined with higher differentiation, indicative of genetic isolation and drift. This suggests these populations are currently being impacted by fragmentation, with implications for their long‐term persistence. Low genetic diversity can impede populations' response to environmental changes or exposure to novel diseases, which may be particularly relevant in urban environments, where wildlife exposure to novel pathogens can be increased (Bradley and Altizer 2007).

All four of the populations examined in this study are close to the inner limits of predicted suitable habitat for hedgehogs across the city (Fung et al. 2024; Turner et al. 2022), particularly the Regent's Park and Lee Valley, which were also observed to have the lowest genetic diversity. Whilst Barnes, which also had low genetic diversity, is located further from the city centre, it is also bordered on three sides by a bend in the River Thames, which may impact gene flow. Interestingly, Hampstead Heath possessed higher genetic diversity, comparable to levels seen outside of the city. As a substantial greenspace in north London, bordered by suburbs to the north, Hampstead Heath and surrounding areas may support a sizeable contiguous hedgehog population. Therefore, these results indicate that genetic isolation due to fragmentation is primarily a risk at high urbanisation pressure, where it could be a significant threat.

In addition to current pressures, observed differences in genetic diversity between the populations may also reflect different histories of the urban environments in which they are found. Welwyn Garden City, for example, possesses particularly high allelic richness, and is a relatively recent settlement constructed in 1920 designed to incorporate greenspace corridors (Pastor et al. 2023), which may be beneficial for maintaining a large and connected local hedgehog population. In contrast, the Regent's Park, which exhibited the lowest genetic diversity and highest differentiation, was enclosed as a park, landscaped, and built around during the 1830s, almost a hundred years earlier (The Royal Parks 2014), and has since faced urban expansion and densification of surrounding infrastructure as Greater London expanded.

Evidence of genetic differentiation in highly urban hedgehog populations has also been found in Barcelona, Spain, where a hedgehog population occupying a greenspace and zoo close to the city centre was found to be genetically differentiated from hedgehogs in the surrounding city, although it was not the least genetically diverse population in the study region (Araguas et al. 2022). Whilst the authors found some evidence of immigration into the population, there was no evidence of emigration, which led the authors to suggest that the highly favourable zoo environment may contribute to genetic isolation by discouraging movement of individuals into less favourable surrounding areas (Araguas et al. 2022).

In Berlin, the presence of local ‘gamodemes’, or kinship networks, of hedgehogs within two large city parks was also attributed to the ease of movement within the parks but their relative isolation from the wider city, for example, due to fencing, a river and large roads (Barthel et al. 2020). However, these populations did not show reduced genetic diversity, and hedgehogs were found to maintain a single connected population across the city, in contrast to the results of this study in Greater London.

### 5.4 Landscape Connectivity

Within Greater London, genetic differentiation among individuals was explained more by isolation through resistance than through distance, with the empirically parameterised resistance surface performing best in least cost path models, and in random walk analysis approaches when distance was included in the model (Table 4). This suggests some consistency in hedgehogs' movement responses to urban landscapes in both Zurich and Greater London. In Zurich, greenspaces were found to facilitate hedgehog movement, particularly private gardens, cemeteries, and allotments, followed by less favoured greenspace, such as amenity grounds and lawns (Braaker et al. 2017). Similarly, greenspace was also identified as a significant corridor for hedgehogs in the single landcover model within Greater London, but only by the commute distance approach. These results support previous findings that greenspace is important for urban hedgehog connectivity, with gardens and allotments, for example, contributing 75% to connectivity in Braunschweig, Germany (App et al. 2022), and with radio tracking studies identifying hedgehog preference for gardens and avoidance of roads and impervious cover (Dowding et al. 2010; Gazzard et al. 2022; Rondinini and Doncaster 2002).

Interestingly, the single landcover models also identified railways as potential corridors for hedgehog gene flow both in Greater London and outside the city. Railways are often considered barriers for wildlife movement; for example, a highly used railway line was found to divide red fox ( *Vulpes vulpes* ) subpopulations in Sapporo, Japan (Kato et al. 2017). Yet red foxes in Berlin and coyotes ( *Canis latrans* ) in Toronto were found to use railways as corridors for movement in urban areas (Gelmi‐Candusso et al. 2024; Kimmig et al. 2020), potentially as the lineside environments are less disturbed by human presence than more publicly accessible greenspaces (Kimmig et al. 2020). Hedgehogs may also avoid high human disturbance, as was for example observed by their increasing movement in response to a temporary music festival in southeast Berlin, Germany (Berger et al. 2020). In addition, as a species associated with habitat edges, such as hedgerows and field margins, hedgehogs may be able to use linear vegetated verges alongside railway tracks to move through urban landscapes.

The empirical resistance model performed poorly outside of Greater London, where isolation by distance was the better predictor of genetic differentiation. This suggests that the processes impacting hedgehog movement differ between highly urbanised and less urban contexts. Interestingly, the single landcover models suggested that farmland may be a potential corridor for movement, rather than a barrier as expected. Before the recent population declines, hedgehogs were predominantly associated with rural landscapes (Yarnell and Pettett 2020), where they moved along field margins.

In combination with the limited presence of clear population clusters identified in the Bayesian clustering analysis, these results suggest that hedgehogs can maintain connected populations across large scales in more rural landscapes, in contrast to urban areas. This is also supported by recent findings that found hedgehogs were able to maintain gene flow in suburban towns separated by agricultural land at local scales in a landscape in rural Nottinghamshire (Yu et al. 2025) and at broader scales which identified limited genetic structuring across South Wales (Shove et al. 2025). Further investigation to understand the role of railways and farmland in facilitating hedgehog dispersal, as well as other urban mammal species, is needed.

## 6 Limitations

Inferences in this study may have been constrained by small sample sizes for each population. However, genomic approaches are often robust to smaller sample sizes and have been suggested to require few individuals per population for accurate inferences (Prunier et al. 2013). Furthermore, the results may be impacted by the long sampling period of 10 years (2012–2022), particularly as there is evidence of hedgehog decline across the study region (Wembridge et al. 2022), and so populations may not be genetically stable over this period.

This study was also limited in that the population level analysis does not include a completely rural population, as the samples sourced from rural locations within this study were widely distributed. Therefore, comparison is between highly urban areas inside Greater London and moderate, more suburban areas outside the city. Therefore, whilst the genetic diversity of populations outside of the city was higher than that inside Greater London, it is not known to what extent these may also be impacted by fragmentation in comparison to rural areas. Use of a greater number of populations from a wider range of more systematically selected sites could be used to provide more detailed insights in future.

Furthermore, the outputs of connectivity analyses can vary widely with different parameterisations of resistance layers (Braaker et al. 2017). Although this study drew on empirical resistance values from the literature, factors other than landcover that can also impact species movement, such as disturbance, vehicular traffic, or interspecific interactions, were not considered which may impact the results. One least‐cost path, for example, followed a route through the busy central core of Greater London (Figure S4). In this study, only a single multi‐category landscape resistance model was used, based on values from previous studies (App et al. 2022; Braaker et al. 2017). High resistance was applied to railways and farmland, whereas single landcover models indicate that this is not accurate for hedgehogs in the study area, and an arbitrary value was chosen for unclassified land uses, which may also miss important habitat variation. When reversing the values of railways and farmland to a value of 1, to put both as corridors for movement in the empirical resistance surface, model outputs surprisingly did not improve and were instead inferior to the null model. This reflects the limitations of examining variables in single landcover models and the need to interpret these results with caution. In future, use of more complex algorithms to extensively and objectively parameterise and optimise land cover resistance values, such as ResistanceGA (Peterman 2018) and Radish (Pope and Peterman 2020), would be valuable to overcome these limitations.

## 7 Conservation Implications

Population viability analysis of hedgehogs previously suggested that a minimum of 90 ha supporting 32–60 hedgehogs is needed to be viable in urban landscapes (Moorhouse 2013). However, this study shows that connectivity between habitats is also important. The Regent's Park and adjacent Primrose Hill, for example, cover 191 ha (The Royal Parks 2014) but are potentially highly isolated, as The Regent's Park hedgehog population possessed only 62%–65% of the genetic diversity observed in populations outside of the city. Indeed, the population is known to have undergone a demographic bottleneck with population estimates of around 10 individuals in recent years (Gurnell et al. 2024). Two other Greater London populations (Barnes and Lee Valley) also showed evidence of genetic diversity loss, indicating that hedgehogs may be vulnerable to fragmentation across the city.

Very low immigration is sufficient to counteract genetic drift and inbreeding depression in fragmented populations (Lacy 1987); therefore, small increases in permeability could have large beneficial impacts. Furthermore, maintaining metapopulation dynamics, in which multiple subpopulations persist and exchange individuals through dispersal (Woodruff 2001), can maintain higher genetic diversity overall in a single panmictic population (Lacy 1987). In this way, these results highlight the value of considering genetic processes in conservation, as approaches that aim to maximise the connectivity of landscapes may be important for maintaining diverse urban terrestrial mammal communities in the future.

## 8 Conclusion

Overall, this study presents important evidence that hedgehog populations occupying urban environments such as cities are vulnerable to genetic isolation due to habitat fragmentation and the challenge of moving through the urban landscape. Our findings suggest that populations in Greater London could be at risk of decline due to genetic processes and highlight the need to also investigate the status of populations in other urban landscapes. Critically, these results suggest that conservation actions to increase habitat connectivity in urban environments will be important for improving the long‐term viability of these vulnerable populations.

## Author Contributions

**Jessica Turner:** conceptualization (equal), data curation (lead), formal analysis (lead), investigation (lead), resources (lead), writing – original draft (lead), writing – review and editing (equal). **Chris Carbone:** conceptualization (equal), investigation (supporting), resources (supporting), supervision (equal), writing – review and editing (equal). **Becki Lawson:** data curation (supporting), resources (supporting), writing – review and editing (equal). **Katharina Seilern‐Macpherson:** data curation (supporting), resources (supporting), writing – review and editing (equal). **Chris G. Faulkes:** conceptualization (equal), investigation (supporting), resources (supporting), supervision (equal), writing – review and editing (equal).

## Funding

Funding for the Garden Wildlife Health Project came from the Banister Charitable Trust; Defra and the Welsh Government, the APHA DoWS (Project ED1058); the Esmée Fairbairn Foundation, the Garfield Weston Foundation, and the Universities Federation for Animal Welfare. IoZ staff receive financial support from Research England.

## Disclosure

Benefits Generated: This research addresses a priority concern for the Western European hedgehog Erinaceus europaeus, and results generated have been shared to benefit conservation of this species. The sequence data generated in the study will be made publicly available to support future research.

## Conflicts of Interest

The authors declare no conflicts of interest.

## Supporting information


**Table S1:** Number of Single Nucleotide Polymorphisms (SNPs) remaining after each bioinformatic filtering step.
**Figure S1:** Post filtering Single Nucleotide Polymorphism (SNP) visualization.
**Table S2:** Table showing landcover classifications used to construct resistance layers.
**Methods S1**. Details of coancestry simulations to identify highly related individuals.
**Figure S2:** Pairwise relatedness plots calculated using Queller and Goodnight estimator.
**Figure S3:** Evanno plots.
**Table S3:** Middle Super Output Areas (MSOA) Population densities for sample sites.
**Figure S4:** Spatial connectivity map for Western European hedgehog *Erinaceus europaeus* in greater London for the best performing model (EMP).
**Table S4:** Sample information for all samples.

**Data S1:** csv file containing SNP dataset for samples analysed in this study.

## Data Availability

Raw read sequences for samples analysed in this study are deposited in the European Nucleotide Archive under project PRJEB101023 and final SNP genotype data included as Supporting Information. The environmental data used in this study are publicly accessible online from their original sources, listed in Supporting Information Table S2.
