# Supplementary material for: Evidence of Population Fragmentation of the Western European Hedgehog Erinaceus europaeus in Urban Landscapes
Source: Ecol Evol. 2026 Apr 22;16(4):e73489. doi: 10.1002/ece3.73489 (PMC13100887; doi:10.1002/ece3.73489)
Supplement: Supplementary file 1 — Table S1: Number of Single Nucleotide Polymorphisms (SNPs) remaining after each bioinformatic filtering step. Figure S1: Post filtering Single Nucleotide Polymorphism (SNP) visualization. Table S2: Table showing landcover classifications used to construct resistance layers. Methods S1. Details of coancestry simulations to identify highly related individuals. Figure S2: Pairwise relatedness plots calculated using Queller and Goodnight estimator. Figure S3: Evanno plots. Table S3: Middle Super Output Areas (MSOA) Population densities for sample sites. Figure S4: Spatial connectivity map for Western European hedgehog Erinaceus europaeus in greater London for the best performing model (EMP). Table S4: Sample information for all samples. [file ECE3-16-e73489-s002.docx]

**Supplementary Files - Evidence of population fragmentation of the Western European hedgehog *Erinaceus europaeus* in urban landscapes**

**Table S1:** Number of Single Nucleotide Polymorphisms (SNPs) remaining after each bioinformatic filtering step.

| Filtering step | No. SNPs remaining |
| --- | --- |
| Retain SNPs only | 38,022 |
| Mean depth < 5 removed | 38,022 |
| Quality <20 removed | 34,332 |
| Depth < 10 removed | 33,795 |
| Mapping quality < 30 removed | 33,794 |
| Minor Allele Count (MAC) < 3 removed | 6,317 |
| Genotyped for < 95% Individuals | 2,172 |
| Retain only biallelic sites | 2,171 |
| Thin to 1 site per UCE | 1,413 |

**Figure S1:** Post filtering Single Nucleotide Polymorphism (SNP) visualisation. Top) density plots of the proportion of missing data per individual (left) and locus (right). Bottom) histograms of mean sequencing depth per individual (left) and locus (right). Mean values are indicated by the dashed red line. One individual with high missing data (> 0.5) was removed prior to analysis.


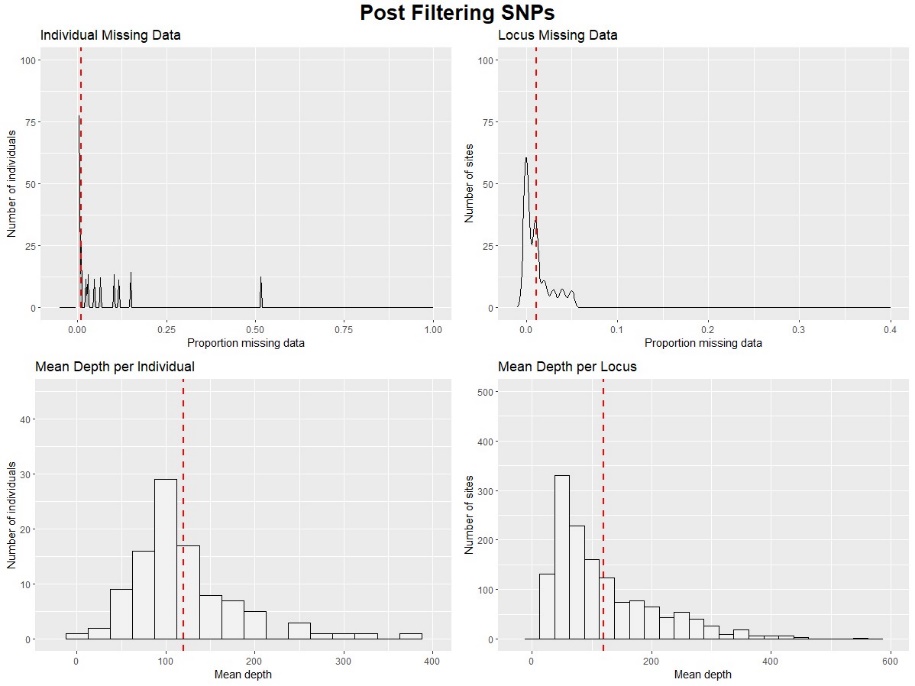


**Table S2:** Table showing landcover classifications used to construct resistance layers for hedgehog movement and online data sources. Source indicates both the dataset and the selected landcover classifications used.

| Landcover | Source |
| --- | --- |
| Parks, Playspace | OS (2021a) Open Greenspace Parks, Playspaces |
| Cemeteries | OS (2021a) Open Greenspace Cemeteries, Religious grounds |
| Private Gardens | OS (2021b) Mastermap Greenspace Private Gardens |
| Allotments | OS (2021a) Open Greenspace Allotments |
| Lawn | OS (2021a) Open Greenspace Playing Field, Bowling Green, Golf Courses |
| Amenity | OS (2021b) Mastermap Greenspace Amenity – Transport, Amenity - Business |
| Woodland | OS (2021c) LocalMap Open – Woodland |
| Wetland | OSM (2023) Natural/ Wetlands |
| Linear Water | OS (2021c) LocalMap Open – Linear Water Features |
| Waterbodies | OS (2021c) LocalMap Open – Surface Area Water, Thames Tidal Water |
| Impervious | OS (2021a) Open Greenspace Tennis, Other Sports.  OSM (2023) Parking |
| Path | OS (2021c) LocalMap Open Shared Use Carriageway, Restricted Local Access Roads |
| Small Street | OS (2021c) LocalMap Open Local Road, Local Access Road, Minor Road, Minor Road Collapsed Dual Carriageway |
| Large Street | OS (2021c) LocalMap Open A Road, A Road Collapsed Dual Carriageway, Primary Road, Primary Road Collapsed Dual Carriageway |
| Highways | OS (2021c) LocalMap Open Motorway, Motorway Collapsed Duel Carriageway, Primary Road, Primary Road Collapsed Dual Carriageway |
| Buildings | OS LocalMap Open Buildings |
| Railway | OS LocalMap Open Railway Tracks |
| Farmland | OSM (2023) Farmland |

**Methods S1:** Details of Coancestry simulations to identify highly related individuals in the dataset.

The presence of highly related individuals can bias population genetic inferences (O’Connell et al., 2019; Wang, 2018). Therefore, pairwise relatedness among samples in the dataset was calculated to identify and remove close relatives using the Queller & Goodnight estimator in Coancestry (Wang, 2011), The most appropriate relatedness estimator was determined through simulations, using 100 individuals of each of nine known relatedness categories (five outbred, three inbred) allele frequencies from the empirical dataset, and locus error rates calculated in the R package ‘poppr’ (Kamvar et al., 2014). Genotyping error rate and allelic drop out were unknown and set to zero. The true relatedness values for outbred simulated individuals were sourced from Coancestry (User guide V. 1.0.1 Wang, 2021), and inbred relationships from Hedrick et al. (2014).

Simulations were run for all seven estimators available with default parameters and 1000 bootstrap repeats. Performance was assessed using the R package ‘related’ (Pew et al., 2015), and correlation to the true relatedness values. This identified the Queller and Goodnight estimator as most appropriate for the calculation of pairwise relatedness estimates for the empirical dataset. Pre-determined relatedness thresholds were used to categorise individuals as first order (R ≥ 0.375), second order (0.1875 ≤ R < 0.375), or third order relatives (0.09375 ≤ R < 0.1875) or unrelated (R < 0.09375), as applied in Davidović et al. (2022). Elevated relatedness was identified among samples from one site, The Regent’s Park, which was also highly sampled due to long-term monitoring and collection of deceased individuals from the population since 2012 (n=33). Therefore, a second analysis was run on The Regent’s Park samples and the remaining samples independently to avoid bias in the relatedness estimates for the rest of the dataset (Taylor, 2015).

The dataset was subset to include only the five most recent samples from The Regent’s Park, excluding first degree relative pairs, in order to reduce the effect of biased sampling towards this location on inferences and to align temporally with much of the rest of the dataset, which was sourced between 2020 – 2022.

**References:**

Davidović, S., Marinković, S., Hribšek, I., Patenković, A., Stamenković-Radak, M., & Tanasković, M. (2022). Sex ratio and relatedness in the Griffon vulture (*Gyps fulvus*) population of Serbia. *PeerJ*, *10*, e14477. https://doi.org/10.7717/peerj.14477

Hedrick, P. W., Peterson, R. O., Vucetich, L. M., Adams, J. R., & Vucetich, J. A. (2014). Genetic rescue in Isle Royale wolves: Genetic analysis and the collapse of the population. *Conservation Genetics*, *15*(5), 1111–1121. https://doi.org/10.1007/s10592-014-0604-1

Kamvar, Z. N., Tabima, J. F., & Grünwald, N. J. (2014). *Poppr*: An R package for genetic analysis of populations with clonal, partially clonal, and/or sexual reproduction. *PeerJ*, *2*, e281. https://doi.org/10.7717/peerj.281

O’Connell, K. A., Mulder, K. P., Maldonado, J., Currie, K. L., & Ferraro, D. M. (2019). Sampling related individuals within ponds biases estimates of population structure in a pond‐breeding amphibian. *Ecology and Evolution*, *9*(6), 3620–3636. https://doi.org/10.1002/ece3.4994

Pew, J., Muir, P. H., Wang, J., & Frasier, T. R. (2015). related: An R package for analysing pairwise relatedness from codominant molecular markers. *Molecular Ecology Resources*, *15*(3), 557–561. https://doi.org/10.1111/1755-0998.12323

Taylor, H. R. (2015). The use and abuse of genetic marker‐based estimates of relatedness and inbreeding. *Ecology and Evolution*, *5*(15), 3140–3150. https://doi.org/10.1002/ece3.1541

Wang, J. (2011). coancestry: A program for simulating, estimating and analysing relatedness and inbreeding coefficients. *Molecular Ecology Resources*, *11*(1), 141–145. https://doi.org/10.1111/j.1755-0998.2010.02885.x

Wang, J. (2018). Effects of sampling close relatives on some elementary population genetics analyses. *Molecular Ecology Resources*, *18*(1), 41–54. https://doi.org/10.1111/1755-0998.12708

**Figure S2:** Pairwise relatedness plots calculated using Queller & Goodnight estimator for A) all successfully sequenced samples (n = 100), B) with samples from The Regents Park (RP) excluded (n = 67), and C) for The Regents Park samples only (n = 33).


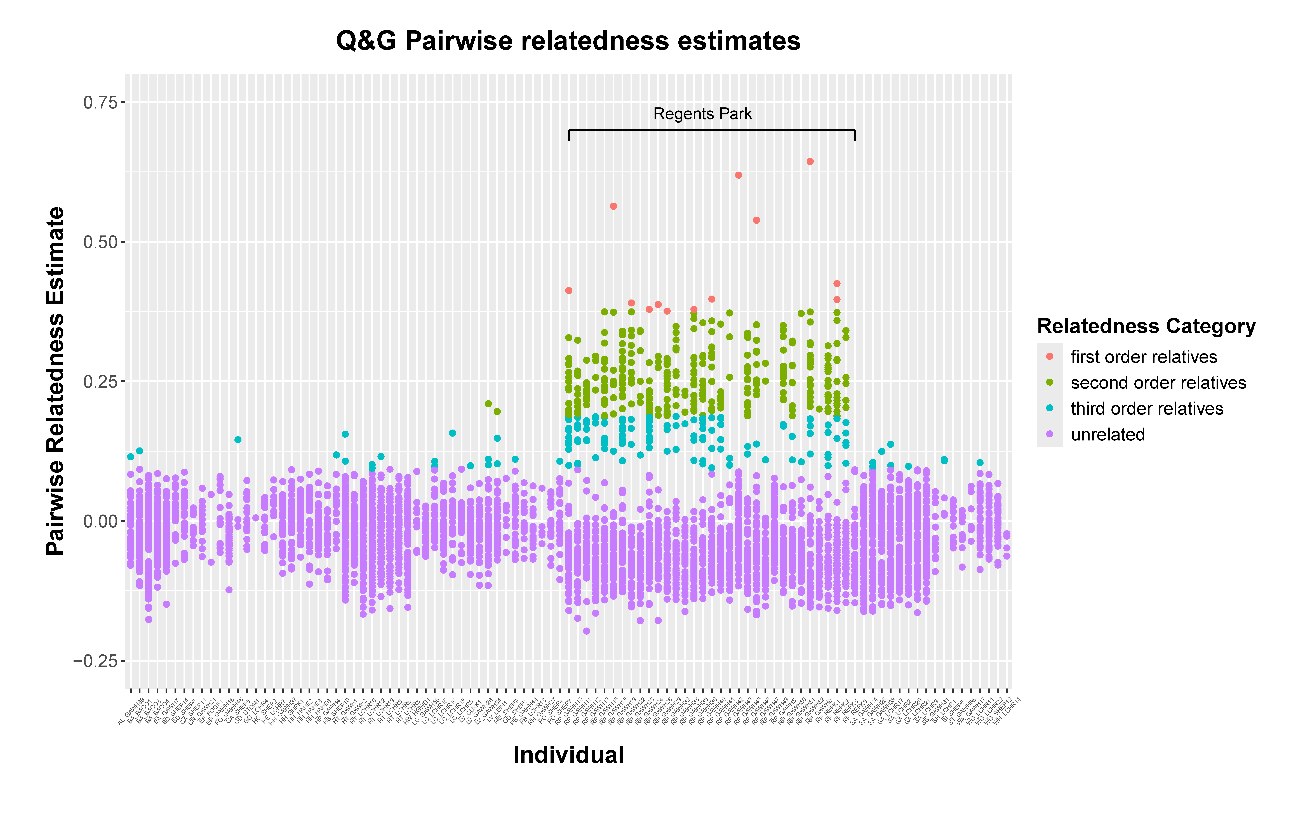
A)


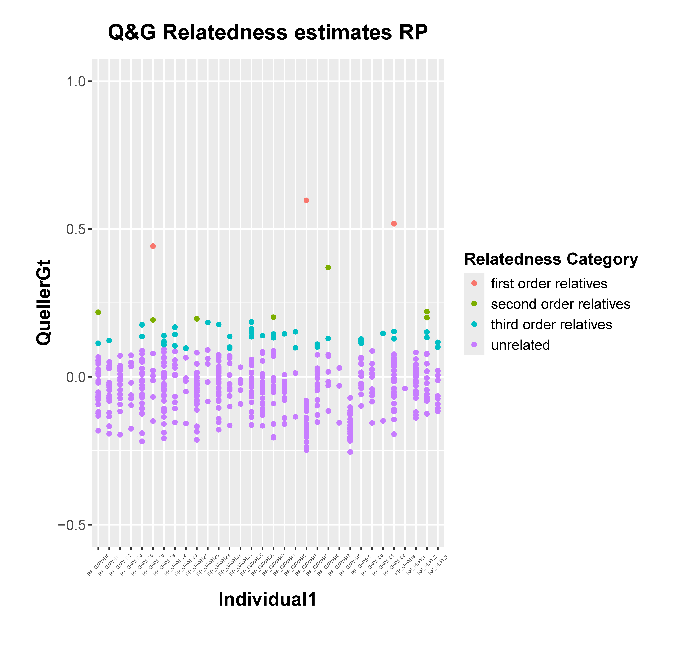

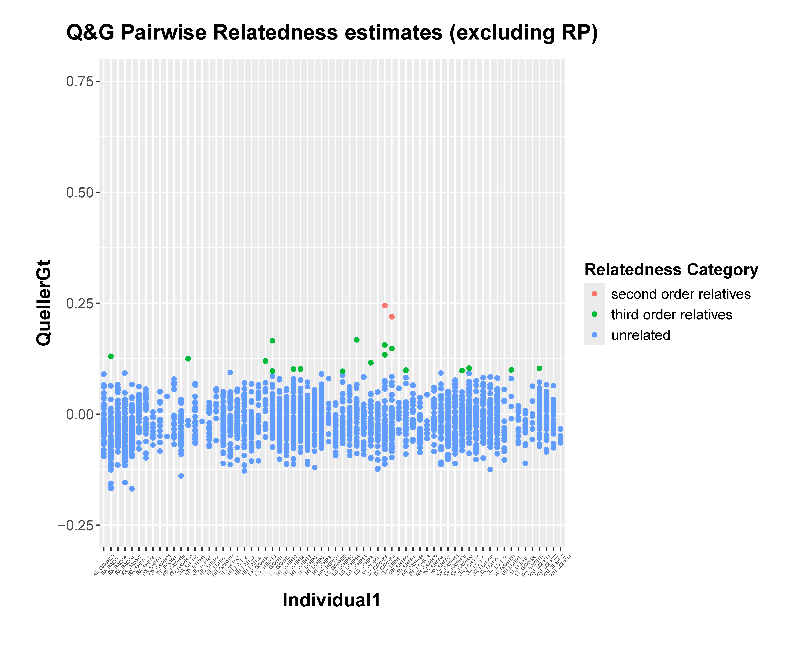
B) C)

**Figure S3:** Evanno Plots – A) Shows the Evanno plot for the full unrelated dataset (n = 72), which supports k = 2 clusters. B) Shows the Evanno plot for the unrelated dataset excluding The Regent’s Park (n = 67) which supports K = 7 clusters.


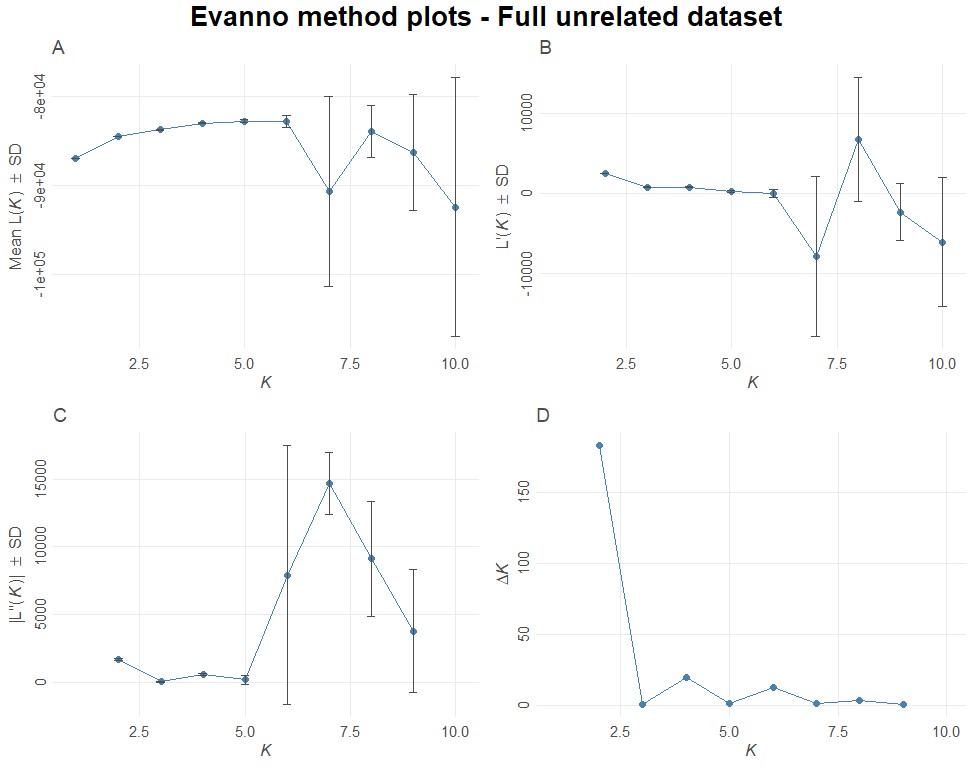
A)


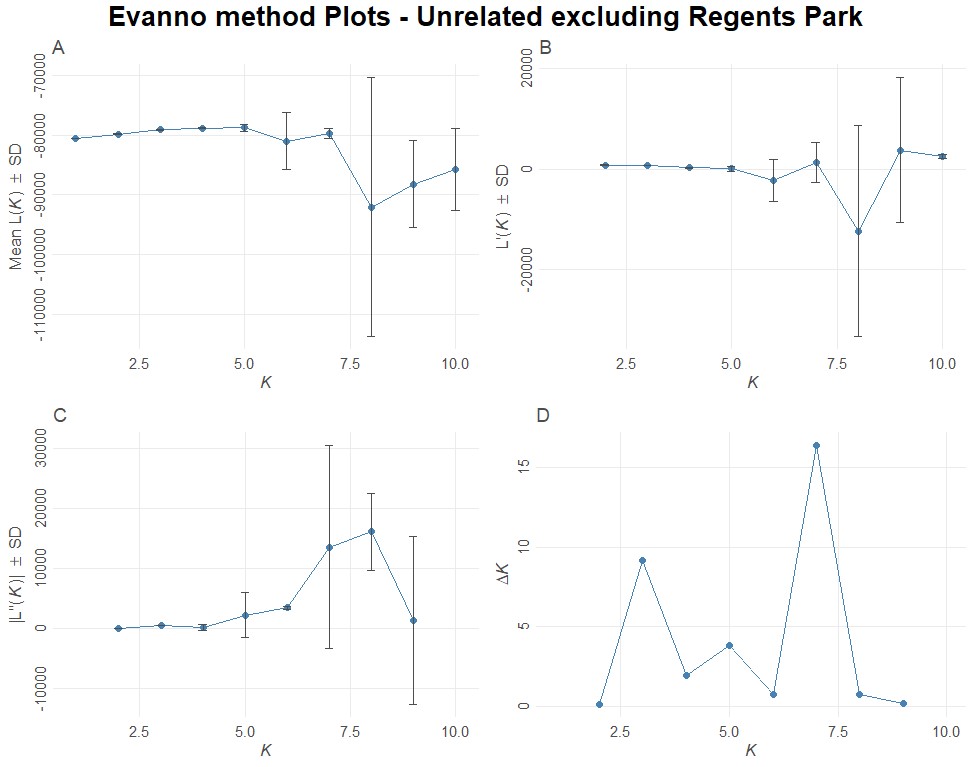
B)

**Table S3:** Middle Super Output Area (MSOA) Population Densities for sample sites. Calculation of mean population densities for sample sites was calculated in order to provide an indication of the urban pressure at each site for comparison. Persons per square kilometre data was accessed at the MSOA level from ONS Census Maps 2021. MSOAs which overlapped the built area of each town were used to calculate a mean population density for the four town sites outside of Greater London. For the four sites within Greater London, the MSOAs in which the samples were located was used. In addition, for Hampstead Heath and The Regent’s Park where samples were all within a greenspace, the MSOAs with boundaries overlapping or adjacent to the greenspace site were also considered.

| Site | MSOA Name | Population Density (persons / km2) |
| --- | --- | --- |
| Barnes | North Barnes | 3837 |
|  | Mortlake and South Barnes | 6171 |
|  | Putney Embankment and Lower Common | 8897 |
| Lee Valley | Markhouse and St James Park | 3897 |
|  | Lea Bridge | 4753 |
|  | Walthamstow Market and South Grove | 14557 |
|  | Blackhorse Road | 5478 |
|  | Stamford Hill South | 14511 |
|  | Upper Clapton | 17508 |
|  | Clapton Leaside | 16184 |
| Regents Park | Regent’s Park | 4283 |
|  | Marylebone and park lane | 6307 |
|  | Church Street | 23792 |
|  | St Johns Wood South | 11101 |
|  | St Johns Wood North | 6672 |
|  | Swiss Cottage | 8920 |
|  | Primrose Hill | 9835 |
|  | Camden Town and Mornington Crescent | 6202 |
|  | Euston | 14140 |
| Hampstead Heath | Hampstead Town | 3458 |
|  | Highgate West | 2561 |
|  | Tufnell Park West | 11480 |
|  | Kentish Town West | 14951 |
|  | Mansfield Road and Park Hill Road | 14175 |
|  | Fitzjohns & Royal Free | 9622 |
|  | Frognal | 6393 |
|  | Golders Green South | 5258 |
|  | Hampstead Garden Suburb | 2829 |
|  | Highgate Wood | 2912 |
| London Colney | London Colney | 1911 |
| St Albans | The Camp and Cunningham | 2832 |
|  | Cottonmill and Sopwell | 2526 |
|  | Verulam Park | 2014 |
|  | St Albans Central | 7049 |
|  | Townsend and New Greens | 3604 |
|  | Clarence Park | 5219 |
|  | Bernards Heath | 3702 |
|  | Marshalswick | 3571 |
|  | Longacres | 6190 |
| Hatfield | Hatfield South | 5984 |
|  | Hatfield North and West | 2496 |
|  | Hatfield Town | 5833 |
| Welwyn Garden City | Welwyn Garden City Central and Handside | 1930 |
|  | Hollybush | 4078 |
|  | Hall Grove | 3591 |
|  | Peartree | 4780 |
|  | Panshanger | 3117 |
|  | Haldens | 2284 |
|  | Knightsfield | 1934 |

**Figure S4:** Spatial connectivity map for Western European hedgehog *Erinaceus europaeus* in Greater London for the best performing model (EMP). The colour gradient indicates cumulative random walk probability between pairwise samples, with grey indicating low and red high probability of movement. Least cost path routes between all sample pairs are shown in blue. Samples are represented by black circles. The location of populations used in population genetic analyses is indicated by the text abbreviations (HH = Hampstead Heath, LV = Lee Valley, BA = Barnes, RP = The Regent’s Park, see Figure 1C) for population outlines). The River Thames is outlined in black.


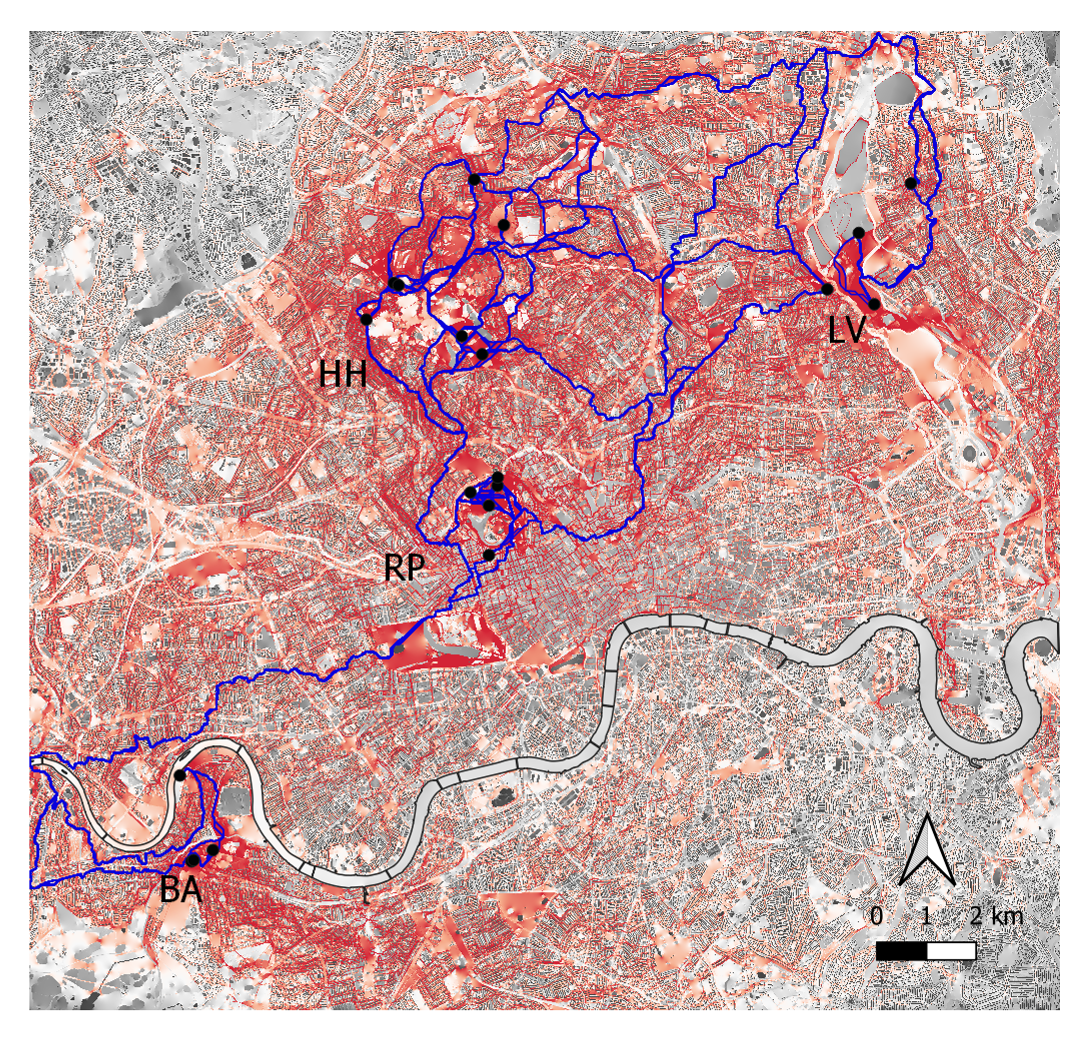


**Contains OS Data © Crown copyright and database right 2021**

**Table S4:** Sample information for all samples (n = 125) including date, coordinates, sex if known, county and assigned locality. Samples which were retained in the final dataset (n = 72) are indicated.

| **Sample** | **Date** | **Latitude** | **Longitude** | **Sex** | **County** | **Locality** | **Final Data** |
| --- | --- | --- | --- | --- | --- | --- | --- |
| GAWH39 | 2018 | 51.701570 | -0.416880 | F | HERTFORDSHIRE | ABBOTS LANGLEY | Y |
| SHEP14 | 24/09/2020 | 51.986300 | -0.197020 | M | HERTFORDSHIRE | BALDOCK | Y |
| SHEP6 | 30/09/2021 | 51.989190 | -0.175380 | F | HERTFORDSHIRE | BALDOCK | Y |
| BACO1 | 22/04/2021 | 51.470194 | -0.238583 | U | GREATER LONDON | BARNES | Y |
| BACO3 | 30/07/2021 | 51.468238 | -0.244868 | U | GREATER LONDON | BARNES | Y |
| BACO4 | 16/11/2021 | 51.468455 | -0.244239 | U | GREATER LONDON | BARNES | Y |
| GAWH7 | 2014 | 51.483864 | -0.247614 | M | GREATER LONDON | BARNES | Y |
| SHEP13 | 22/09/2021 | 52.357110 | 0.007260 | F | CAMBRIDGESHIRE | BLUNTISHAM | Y |
| SHEP11 | 23/09/2021 | 52.189800 | 0.145260 | F | CAMBRIDGESHIRE | CAMBRIDGE | Y |
| GAWH21 | 2015 | 51.373427 | -0.801774 | M | BERKSHIRE | CROWTHORNE | Y |
| FOGR1 | 28/10/2021 | 51.590586 | -0.157902 | U | GREATER LONDON | EAST FINCHLEY | Y |
| GAWH16 | 2015 | 51.693140 | 0.118184 | M | ESSEX | EPPING | Y |
| GAWH48 | 2019 | 51.084198 | 1.189866 | F | KENT | FOLKSTONE | Y |
| SHEP5 | 21/08/2021 | 52.251400 | 0.515990 | F | SUFFOLK | GAZELEY | Y |
| LCHR4 | 21/06/2021 | 51.708046 | -0.097335 | F | HERTFORDSHIRE | GOFFS OAK | Y |
| GTDM1 | 26/06/2021 | 51.875070 | 0.350230 | U | ESSEX | GREATER DUNMOW | Y |
| GAWH37 | 2018 | 51.558873 | -0.156917 | M | GREATER LONDON | HAMPSTEAD HEATH | Y |
| GHPK1 | 25/09/2020 | 51.565688 | -0.190315 | U | GREATER LONDON | HAMPSTEAD HEATH | Y |
| HAHE2 | 22/07/2020 | 51.562250 | -0.162690 | U | GREATER LONDON | HAMPSTEAD HEATH | Y |
| HAHE3 | 27/05/2021 | 51.571763 | -0.180696 | U | GREATER LONDON | HAMPSTEAD HEATH | Y |
| HAHE6 | 05/08/2022 | 51.572201 | -0.182027 | U | GREATER LONDON | HAMPSTEAD HEATH | Y |
| GAWH4 | 2013 | 51.826599 | -0.354454 | M | HERTFORDSHIRE | HARPENDEN | Y |
| GAWH1 | 2012 | 51.752807 | -0.226746 | F | HERTFORDSHIRE | HATFIELD | Y |
| GAWH2 | 2012 | 51.752807 | -0.226746 | M | HERTFORDSHIRE | HATFIELD | Y |
| LCHR10 | 25/06/2021 | 51.751909 | -0.233001 | M | HERTFORDSHIRE | HATFIELD | Y |
| LCHR12 | 23/06/2021 | 51.774125 | -0.239868 | F | HERTFORDSHIRE | HATFIELD | Y |
| LCHR19 | 01/07/2021 | 51.767837 | -0.247748 | U | HERTFORDSHIRE | HATFIELD | Y |
| LCHR5 | 16/06/2021 | 51.769334 | -0.225652 | F | HERTFORDSHIRE | HATFIELD | Y |
| LCHR6 | 18/06/2021 | 51.772298 | -0.211957 | M | HERTFORDSHIRE | HATFIELD | Y |
| LCHR8 | 07/05/2021 | 51.766993 | -0.229992 | M | HERTFORDSHIRE | HATFIELD | Y |
| LCHR7 | 23/06/2021 | 51.764483 | -0.465270 | F | HERTFORDSHIRE | HEMEL HEMPSTEAD | Y |
| HGWD1 | 22/07/2022 | 51.582190 | -0.149670 | U | GREATER LONDON | HIGHGATE WOODS | Y |
| SHEP10 | 31/07/2021 | 52.251260 | 0.101550 | F | CAMBRIDGESHIRE | HISTON | Y |
| SHEP2 | 28/08/2021 | 51.949665 | -0.284792 | F | HERTFORDSHIRE | HITCHIN | Y |
| CLHK1 | 11/08/2021 | 51.569036 | -0.056200 | U | GREATER LONDON | LEE VALLEY | Y |
| GAWH26 | 2016 | 51.579095 | -0.046610 | F | GREATER LONDON | LEE VALLEY | Y |
| GAWH34 | 2017 | 51.587828 | -0.031078 | M | GREATER LONDON | LEE VALLEY | Y |
| LERI1 | 15/09/2021 | 51.566091 | -0.042676 | U | GREATER LONDON | LEE VALLEY | Y |
| GAWH55 | 2015 | 51.726915 | -0.300158 | F | HERTFORDSHIRE | LONDON COLNEY | Y |
| LCHR15 | 26/06/2021 | 51.726181 | -0.308940 | U | HERTFORDSHIRE | LONDON COLNEY | Y |
| LCHR16 | 30/06/2021 | 51.729615 | -0.301414 | M | HERTFORDSHIRE | LONDON COLNEY | Y |
| LCHR18 | 02/07/2021 | 51.725238 | -0.303903 | M | HERTFORDSHIRE | LONDON COLNEY | Y |
| LCHR3 | 19/06/2021 | 51.721077 | -0.301258 | F | HERTFORDSHIRE | LONDON COLNEY | Y |
| SHEP9 | 15/08/2021 | 52.093335 | 0.004930 | M | CAMBRIDGESHIRE | MELDRETH | Y |
| SHEP1 | 09/06/2021 | 52.027890 | -0.110020 | M | CAMBRIDGESHIRE | ODSEY | Y |
| GAWH44 | 2019 | 51.706760 | -0.156180 | F | HERTFORDSHIRE | POTTERS BAR | Y |
| LCHR13B | 26/06/2021 | 51.691193 | -0.175157 | F | HERTFORDSHIRE | POTTERS BAR | Y |
| GAWH27 | 2016 | 51.257192 | -0.159892 | F | SURREY | REDHILL | Y |
| GAWH43 | 2019 | 51.531407 | -0.156065 | F | GREATER LONDON | REGENTS PARK | Y |
| GAWH50 | 2020 | 51.534970 | -0.153443 | M | GREATER LONDON | REGENTS PARK | Y |
| GAWH51 | 2020 | 51.522420 | -0.156428 | F | GREATER LONDON | REGENTS PARK | Y |
| REPK3 | 02/11/2021 | 51.536484 | -0.153301 | M | GREATER LONDON | REGENTS PARK | Y |
| REPK1 | 18/07/2020 | 51.533888 | -0.161341 | U | GREATER LONDON | REGENTS PARK | Y |
| SHEP4 | 23/08/2021 | 52.057300 | -0.033590 | F | HERTFORDSHIRE | ROYSTON | Y |
| SHEP7 | 08/03/2021 | 52.057620 | -0.023720 | U | HERTFORDSHIRE | ROYSTON | Y |
| LCHR1 | 16/06/2021 | 51.695231 | -0.293668 | F | HERTFORDSHIRE | SHENLEY | Y |
| GAWH14 | 2015 | 51.771851 | -0.298470 | M | HERTFORDSHIRE | ST ALBANS | Y |
| GAWH25 | 2016 | 51.749695 | -0.321033 | M | HERTFORDSHIRE | ST ALBANS | Y |
| GAWH38 | 2018 | 51.753877 | -0.299146 | M | HERTFORDSHIRE | ST ALBANS | Y |
| LCHR17 | 29/06/2021 | 51.752962 | -0.329824 | M | HERTFORDSHIRE | ST ALBANS | Y |
| LCHR2 | 17/06/2021 | 51.752856 | -0.311638 | F | HERTFORDSHIRE | ST ALBANS | Y |
| LCHR20 | 02/07/2021 | 51.737384 | -0.344211 | F | HERTFORDSHIRE | ST ALBANS | Y |
| LCHR21 | 03/07/2021 | 51.756976 | -0.325736 | U | HERTFORDSHIRE | ST ALBANS | Y |
| LCHR9 | 18/09/2021 | 51.757725 | -0.338696 | M | HERTFORDSHIRE | ST ALBANS | Y |
| SHEP8 | 26/09/2021 | 52.336560 | -0.080190 | M | CAMBRIDGESHIRE | ST IVES | Y |
| GAWH56 | 2015 | 51.366576 | -0.256072 | M | SURREY | STONELEIGH | Y |
| GAWH31 | 2017 | 51.385176 | -0.298479 | F | GREATER LONDON | SURBITON | Y |
| GAWH17 | 2015 | 51.683002 | -0.374131 | F | HERTFORDSHIRE | WATFORD | Y |
| LCHR11 | 20/06/2021 | 51.792735 | -0.184990 | U | HERTFORDSHIRE | WELWYN GARDEN CITY | Y |
| LCHR22A | 02/07/2021 | 51.782654 | -0.199683 | F | HERTFORDSHIRE | WELWYN GARDEN CITY | Y |
| SHEP12 | 13/06/2021 | 51.801750 | -0.161810 | M | HERTFORDSHIRE | WELWYN GARDEN CITY | Y |
| LCHR14 | 20/06/2021 | 51.636455 | -0.178386 | F | GREATER LONDON | WHETSTONE | Y |
| GAWH10 | 2014 | 51.532583 | -0.155582 | F | GREATER LONDON | REGENTS PARK |  |
| GAWH11 | 2014 | 51.532593 | -0.155050 | M | GREATER LONDON | REGENTS PARK |  |
| GAWH12 | 2014 | 51.532763 | -0.150804 | F | GREATER LONDON | REGENTS PARK |  |
| GAWH13 | 2015 | 51.531407 | -0.156065 | M | GREATER LONDON | REGENTS PARK |  |
| GAWH15 | 2015 | 51.536107 | -0.154270 | F | GREATER LONDON | REGENTS PARK |  |
| GAWH18 | 2015 | 51.522420 | -0.156428 | M | GREATER LONDON | REGENTS PARK |  |
| GAWH19 | 2015 | 51.536596 | -0.149246 | F | GREATER LONDON | REGENTS PARK |  |
| GAWH22 | 2016 | 51.531407 | -0.156065 | F | GREATER LONDON | REGENTS PARK |  |
| GAWH23 | 2016 | 51.531407 | -0.156065 | M | GREATER LONDON | REGENTS PARK |  |
| GAWH24 | 2016 | 51.522420 | -0.156428 | M | GREATER LONDON | REGENTS PARK |  |
| GAWH28 | 2017 | 51.522420 | -0.156428 | M | GREATER LONDON | REGENTS PARK |  |
| GAWH29 | 2017 | 51.529166 | -0.164482 | F | GREATER LONDON | REGENTS PARK |  |
| GAWH32 | 2017 | 51.531407 | -0.156065 | F | GREATER LONDON | REGENTS PARK |  |
| GAWH33 | 2017 | 51.525435 | -0.155762 | F | GREATER LONDON | REGENTS PARK |  |
| GAWH35 | 2018 | 51.531407 | -0.156065 | F | GREATER LONDON | REGENTS PARK |  |
| GAWH36 | 2018 | 51.522420 | -0.156428 | F | GREATER LONDON | REGENTS PARK |  |
| GAWH40 | 2018 | 51.535204 | -0.153922 | F | GREATER LONDON | REGENTS PARK |  |
| GAWH41 | 2019 | 51.531407 | -0.156065 | F | GREATER LONDON | REGENTS PARK |  |
| GAWH42 | 2019 | 51.531407 | -0.156065 | F | GREATER LONDON | REGENTS PARK |  |
| GAWH45 | 2019 | 51.531407 | -0.156065 | M | GREATER LONDON | REGENTS PARK |  |
| GAWH46 | 2019 | 51.531407 | -0.156065 | F | GREATER LONDON | REGENTS PARK |  |
| GAWH47 | 2019 | 51.522420 | -0.156428 | M | GREATER LONDON | REGENTS PARK |  |
| GAWH49 | 2019 | 51.531407 | -0.156065 | F | GREATER LONDON | REGENTS PARK |  |
| GAWH5 | 2013 | 51.536534 | -0.148904 | F | GREATER LONDON | REGENTS PARK |  |
| GAWH52 | 2020 | 51.522420 | -0.156428 | M | GREATER LONDON | REGENTS PARK |  |
| GAWH9 | 2014 | 51.526451 | -0.149036 | M | GREATER LONDON | REGENTS PARK |  |
| REPK2 | 2020 | 51.534294 | -0.158192 | U | GREATER LONDON | REGENTS PARK |  |
| REPK5 |  | 51.531407 | -0.156065 | U | GREATER LONDON | REGENTS PARK |  |
| BUPK1 | 23/08/2021 | 51.412549 | -0.353048 | U | GREATER LONDON | BUSHY |  |
| BUPK2 | 23/08/2021 | 51.410936 | -0.352939 | U | GREATER LONDON | BUSHY |  |
| BUPK3 | 07/09/2021 | 51.416926 | -0.326408 | U | GREATER LONDON | BUSHY |  |
| CHHS1 | 29/04/2021 | 51.485473 | -0.263588 | U | GREATER LONDON | CHISWICK |  |
| FIAL1 | 02/12/2020 | 51.609109 | -0.228270 | U | GREATER LONDON | FINCHLEY |  |
| HAHE1 | 18/09/2020 | 51.564104 | -0.169940 | U | GREATER LONDON | HAMPSTEAD HEATH |  |
| HAHE5 | 05/08/2022 | 51.557965 | -0.168375 | U | GREATER LONDON | HAMPSTEAD HEATH |  |
| GAWH3 | 2013 | 51.537904 | -0.144886 | F | GREATER LONDON | REGENTS PARK |  |
| WNFT1 | 15/09/2021 | 51.561004 | 0.028577 | U | GREATER LONDON | WANSTEAD FLATS |  |
| BAAL1 | 06/06/2021 | 51.482271 | -0.234987 | U | GREATER LONDON | BARNES |  |
| BACO2 | 15/05/2021 | 51.469788 | -0.237361 | U | GREATER LONDON | BARNES |  |
| GAWH6 | 2014 | 51.263158 | -0.589651 | M | SURREY | GUILDFORD |  |
| GAWH53 | 2015 | 51.761574 | -0.211912 | F | HERTFORDSHIRE | HATFIELD |  |
| LUTO1 | 13/02/2020 | 51.877495 | -0.385191 | U | BEDFORDSHIRE | LUTON |  |
| LCHR13A | 26/06/2021 | *51.691193* | *-0.175157* | F | HERTFORDSHIRE | POTTERS BAR |  |
| GAWH20 | 2015 | 51.530966 | -0.164888 | M | GREATER LONDON | REGENTS PARK |  |
| GAWH30 | 2017 | 51.530924 | -0.146268 | M | GREATER LONDON | REGENTS PARK |  |
| REPK4 | 14/07/2021 | 51.533941 | -0.161181 | U | GREATER LONDON | REGENTS PARK |  |
| GAWH8 | 2014 | 51.120043 | 0.849259 | F | KENT | STUBBS CROSS |  |
| GAWH54 | 2014 | 51.435541 | -0.066409 | M | GREATER LONDON | SYDENHAM |  |
| LCHR22B | 02/07/2021 | *51.782654* | *-0.199683* | *U* | HERTFORDSHIRE | WELWYN GARDEN CITY |  |
| BUPK4 | 29/03/2022 |  |  | U |  |  |  |
| HAHE4 | 04/07/2022 |  |  | U |  |  |  |
| KNWD1 | 31/07/2020 |  |  | U |  |  |  |
| SHEP3 | 31/01/2021 |  |  | U |  |  |  |
